# Sex differences in the human reward system: convergent behavioral, autonomic and neural evidence

**DOI:** 10.1093/scan/nsaa104

**Published:** 2020-07-30

**Authors:** Katherine G Warthen, Alita Boyse-Peacor, Keith G Jones, Benjamin Sanford, Tiffany M Love, Brian J Mickey

**Affiliations:** 1 Department of Psychiatry, University of Utah, Salt Lake City, UT 84108, USA; 2 College of Arts and Sciences, Oberlin College, Oberlin, OH 44074, USA; 3 Department of Psychiatry, University of Michigan, Ann Arbor, MI 48109, USA

**Keywords:** sex differences, reward, salience, fMRI, motivation

## Abstract

Several studies have suggested that females and males differ in reward behaviors and their underlying neural circuitry. Whether human sex differences extend across neural and behavioral levels for both rewards and punishments remains unclear. We studied a community sample of 221 young women and men who performed a monetary incentive task known to engage the mesoaccumbal pathway and salience network. Both stimulus salience (behavioral relevance) and valence (win *vs* loss) varied during the task. In response to high- *vs* low-salience stimuli presented during the monetary incentive task, men showed greater subjective arousal ratings, behavioral accuracy and skin conductance responses (*P* < 0.006, Hedges’ effect size *g* = 0.38 to 0.46). In a subsample studied with functional magnetic resonance imaging (*n* = 44), men exhibited greater responsiveness to stimulus salience in the nucleus accumbens, midbrain, anterior insula and dorsal anterior cingulate cortex (*P* < 0.02, *g* = 0.86 to 1.7). Behavioral, autonomic and neural sensitivity to the valence of stimuli did not differ by sex, indicating that responses to rewards *vs* punishments were similar in women and men. These results reveal novel and robust sex differences in reward- and punishment-related traits, behavior, autonomic activity and neural responses. These convergent results suggest a neurobehavioral basis for sexual dimorphism observed in the reward system, including reward-related disorders.

## Introduction

Sex differences in the brain have increasingly been the subject of scientific and social debate. A National Institutes of Health policy requiring the consideration of sex differences in biomedical research has recently brought sexual dimorphism of the brain into the spotlight (reference to NIH policy). Numerous studies of humans and other animals have reported differences in brain structure and function between the sexes (Pohjalainen *et al.,* 1998; Andersen and Teicher, 2000; Lavalaye *et al.,* 2000; Sarton *et al.,* 2000; Mozley *et al.,* 2001; Staley *et al.,* 2001; Laakso *et al.,* 2002; Lynch *et al.,* 2002; Cepeda and Carr, 2003; Russo *et al.,* 2003; Cahill, 2006; Mcarthur *et al.,* 2007; Ingalhalikar *et al.,* 2014; Ruigrok *et al.,* 2014; Hausmann, 2017), but the meaning and behavioral impact of these sex differences remains controversial (Eliot and Richardson, 2016; Eliot, 2019; Voskuhl and Klein, 2019).

Psychiatric disorders involving the reward system often present differently in men and women (Becker *et al.,* 2017). For example, men are more likely to participate in activities with a high risk of addiction, such as gambling or drug abuse, but women who participate in these activities may be more sensitive to drug effects and escalate to misuse more rapidly (Fattore *et al.,* 2014; Becker, 2016; Riley *et al.,* 2018; Mayo *et al.,* 2019). Similarly, major depression is more common in women and may present in a sex-specific manner. For example, depressed men report more symptoms of risk taking and impulsivity, whereas depressed women are more likely to report mood disturbance (Cavanagh *et al.,* 2017). Observations like these indicate that women and men differ in their vulnerability to reward-related disorders. A better understanding of differences in reward behavior and brain function between females and males is expected to provide insight into the underlying pathogenesis of disorders of the reward system. Furthermore, this knowledge may be useful to prevent these illnesses and improve treatment of both men and women.

Studies of the reward system in rodents have demonstrated clear sex differences in behavior and neural function (Perry *et al.,* 2013; Dickson *et al.,* 2015; Becker, 2016; Becker and Koob, 2016). For example, female rats escalate cocaine self-administration more quickly than males, an effect that may depend on estrogen (Lynch *et al.,* 2001; Jackson *et al.,* 2006). Hormone- and drug-specific sex differences are also seen during withdrawal (Carroll and Anker, 2009; Becker and Koob, 2016), where estrogen and progesterone levels can impact drug seeking behavior and negative affect during withdrawal. A recent behavioral study of rodents found that reward-guided learning and cognitive flexibility were similar between males and females, but females learned more rapidly to avoid punishment and were more sensitive to unpredictable negative outcomes (Chowdhury *et al.,* 2019). These behavioral differences are accompanied by neural differences between males and females. For example, females have lower dopamine levels in the nucleus accumbens (NAc), lower striatal D1 receptor expression and a different pattern of striatal activation to amphetamine (Becker, 2016; Becker and Koob, 2016). Thus, studies of animal models have demonstrated neurobehavioral differences between females and males, some of which depend on the valence of incentives (reward *vs* punishment).

Sex differences in the human reward system have been evaluated in previous neuroimaging studies using a variety of methods, and the findings have been mixed. Munro *et al.* (2006) found greater dopamine release in the ventral striatum in response to amphetamine in men compared to women, and a similar sex difference was found in the right ventral striatum during tobacco smoking (Cosgrove *et al.,* 2014). Adolescent boys showed higher NAc response compared to girls in anticipation of monetary gains during a risky decision-making task, and also made riskier decisions (Alarcón *et al.,* 2017). Curtis *et al.* (2019) also reported greater ventral striatum BOLD in men during win trials of a gambling task. In contrast, women showed a greater response than men in the NAc in response to hedonic foods when fasting, but not in a fed state (Legget *et al.,* 2018). Other studies reported no significant sex differences in the NAc during reward tasks (Dreher *et al.,* 2007; Spreckelmeyer *et al.,* 2009; Diekhof *et al.,* 2012; Morgan *et al.,* 2013). Early studies were limited by small sample sizes, and most previous studies did not test negative-valence stimuli (e.g. monetary loss). Analysis of both gain and loss is needed to clarify whether sex differences are specific to rewards or rather generalize to salient stimuli regardless of valence. Furthermore, few previous reports investigating sex differences in the reward system have included task-relevant performance or subjective ratings. Thus, the behavioral impact of any neural sex differences remains largely unknown.

Here we addressed the limitations of previous studies by examining sex differences in reward function in a large community sample of young adults. Our objective was to create a more comprehensive picture of sex differences by examining responses to both positive- and negative-valence cues (rewards and losses) across multiple levels of analysis: subjective ratings of incentive stimuli, task performance, autonomic arousal during the task and neural responses. Subjective ratings provide insight into how men and women differentially perceive incentive stimuli, while task performance represents quantifiable behavior when presented with potential rewards and losses. Autonomic function, measured via skin conductance response (SCR), gives an objective indicator of sex differences in arousal during the task. Neural responses allow one to examine the brain basis of these differences in response to the task between the sexes. We used a version of the monetary incentive delay (MID) task that quantified anticipatory responses and allowed us to distinguish sensitivity to salience (behavioral relevance) *vs* sensitivity to valence (win *vs* loss). This task strongly engages the mesoaccumbal pathway (NAc and midbrain) as well as the broader salience network, which is centered on the dorsal anterior cingulate cortex (dACC) and anterior insula (AI), so those structures were examined as regions of interest (Menon, 2015; Warthen *et al.,* 2018). We also measured sex differences in psychological traits related to reward and punishment sensitivity (Carver and White, 1994; Torrubia *et al.,* 2001; Jackson and Smillie, 2004) to complement these reward task-related metrics, and to evaluate how commonly measured traits map onto behavior. We hypothesized that women would respond less strongly to increases in stimulus salience, and more strongly to negative valence stimuli across multiple neurobehavioral levels of analysis.

## Material and methods

### Design, participants and questionnaires

The study was approved by the University of Michigan Institutional Review Board. A community sample of right-handed adults (*n* = 221) aged 18–22 were enrolled in a protocol that included two visits. Health status was self-reported, and participants were also assessed by a nurse and had vital signs taken. During the first visit, participants completed the informed consent process, the Mini International Neuropsychiatric Interview [MINI, version 5.0.0, (Sheehan *et al.,* 1998)], questionnaires, and skin conductance measurement during a MID task. Further details concerning MINI exclusion criteria and screening are included in the supplement. For all individuals, self-reported sex agreed with genetic sex, as determined by genotyping of blood samples (see Supplementary Methods). Participants completed the Positive and Negative Affect Schedule [PANAS (Watson *et al.,* 1988)] and Center for Epidemiologic Studies Depression Scale [CES-D (Radloff, 1977)] as measures of emotional state. Reward- and punishment-related traits were measured with the Behavioral Inhibition and Approach Scales [BIS-BAS (Carver and White, 1994)], Sensitivity to Punishment and Sensitivity to Reward Questionnaire [SPSRQ (Torrubia *et al.,* 2001)] and Appetitive Motivation Scale [AMS (Jackson and Smillie, 2004)]. To comprehensively measure psychological traits, including those not obviously related to reward measures, subjects completed the NEO Personality Inventory—Revised [NEO-PI-R (Costa Jr. and McCrae, 1995)]. A subset of 53 participants with specific genotypes participated in the second visit which involved functional magnetic resonance imaging (fMRI), and data from 44 were available for analysis after quality-control screening, as described below.

In a previous publication (Warthen *et al.,* 2018), we described the effect of neuropeptide Y (NPY) genotype group on fMRI responses for the subset of 53 imaged subjects. Participants selected for imaging fell into one of two NPY genotype groups. Sex differences were not analyzed in the original publication. Here we report findings from the full sample of 221 subjects across a range of outcomes: psychological traits, task performance, stimulus ratings, SCRs and fMRI responses.

### Behavioral task

A modified version of the MID task (Cooper and Knutson, 2008) was used in this study. This task is well suited to our aims as it allows for independent variation of both valence and salience of stimuli. Details are described in the Supplement and in a previous report (Warthen *et al.,* 2018) and briefly summarized here. The five trial types were: high-salience and positive-valence (uncertain win); high-salience and negative-valence (uncertain loss); low-salience and positive-valence (certain win); low-salience and negative-valence (certain loss) and neutral. At the beginning of each trial, the trial type (condition) was indicated by a cue displayed on a screen (‘W?’, ‘L?’, ‘W’, ‘L’ or ‘N’; ‘W’ and ‘L’ represent win and loss, ‘?’ indicates a salient/uncertain outcome and ‘N’ is neutral). During high-salience trials, participants had the opportunity to win $1 or avoid losing $1 if they performed accurately. On low-salience trials, participants won or lost $1 regardless of performance. No money was at stake during neutral trials. The five conditions were presented in pseudo-random order with 20 repetitions per condition. After the MID task, subjects rated each cue stimulus on arousal and affect (Warthen *et al.,* 2018). For affect, participants rated ‘how positive or negative you feel,’ from 1 (negative) to 5 (positive). Arousal was rated based on ‘how aroused you feel,’ from 1 (‘not aroused’) to 5 (‘very aroused’). All participants performed this task outside of the scanner, and a subset of 53 subjects performed the task during functional MRI. The task was optimized to model neural responses only during the anticipatory phase of the MID task because this is when the largest neural responses to the task occur. By design, the trial duration was as short as possible so that more repetitions of each trial type could be acquired, improving the signal-to-noise ratio, and therefore the target and feedback phases of this task were not variable enough in timing to model independently.

### Skin conductance

SCR was collected as an objective measure of arousal (autonomic function) during performance of the MID task using a Biopac system (MP150; Goleta, CA). Each subject took part in two MID task runs (50 trials each) outside of the MRI scanner. After the quality-control step (see supplement), data from 201 subjects were available for analysis. Mean peak-to-peak SCR (measured in μS) was calculated across 20 repetitions of each task condition. SCR z-scores were calculated as the mean peak-to-peak value divided by the peak-to-peak standard deviation across 20 repetitions of each task condition. Because the distribution of z-scores was skewed, log10 transformed SCR z-values were used for statistical analyses. Further methodological details are provided in the Supplementary Methods.

### Neuroimaging

Task-evoked blood oxygenation-level-dependent (BOLD) T2^*^-weighted data were acquired on a 3-Tesla scanner and images were processed using SPM and custom software, as previously described (Warthen *et al.,* 2018). See supplement for imaging details. Data from 44 subjects were available for analysis after quality-control procedures. Those 44 participants were similar to the remainder of the sample on demographic, physiological and clinical variables (see Supplementary Material and Supplementary Table S1).

### Brain regions of interest

The primary regions of interest in this study were selected a priori based on their known involvement in reward-related behavior and their engagement during the MID task. The mesoaccumbal pathway includes projections of dopaminergic neurons from the midbrain (ventral tegmentum and substantia nigra pars compacta) to the bilateral NAc. In addition to these subcortical structures, the MID task activates the major cortical nodes of the salience network, viz., the dACC and the bilateral AI. These regions were anatomically defined based on independent, previously published data (see Supplementary Methods and Supplementary Figure S1). The AI was divided into dorsal and ventral subregions (Gorgolewski *et al.,* 2015) based on evidence of functional heterogeneity (Chang *et al.,* 2013). BOLD contrast values were extracted from voxel-wise fMRI contrast images, spatially averaged across each region of interest, and analyzed with linear models as described below.

### Statistical analyses

Analyses were performed in R (version 3.4.1). We evaluated five outcome measures related to the MID task: accuracy, arousal ratings, affect ratings, SCRs and fMRI responses. Each response measure was calculated per task condition as the mean value relative to the mean of all neutral trials (high-salience win minus neutral, high-salience loss minus neutral, etc.). Linear mixed models (‘lmer’ function, ‘lme4’ package, version 1.1.21) included subject as a random-effects predictor. Fixed-effect predictors included stimulus salience (high or low), stimulus valence (win or loss) and salience-by-valence interaction. Because imaged subjects fell into one of two predefined genotype groups, and genotype group did influence NAc and midbrain responses [High or Low NPY expression, see Supplementary Methods and (Warthen *et al.,* 2018)], all fMRI analyses additionally included genotype group as a fixed-effect predictor of no interest.

Sex differences were tested with linear mixed models including subject as a random-effects predictor. Fixed-effect predictors included sex, salience, valence and two-way interactions. We also evaluated sex differences with linear models (‘lm’ function). For each outcome measure (accuracy, arousal ratings, affect ratings, SCRs and fMRI responses), the effect of sex was separately analyzed for the salience contrast—defined as the sum of the two uncertain (high salience) conditions minus the sum of the two certain (low salience) conditions—and for the valence contrast—defined as the sum of the two gain conditions minus the sum of the two loss conditions. We calculated Hedges’ g to determine effect sizes of sex differences (‘cohen.d’ function, ‘effsize’ package, version 0.7.6).

**Table 1:** Demographic, physiological and clinical characteristics of the sample

	All (*n* = 221)		Men (*n* = 100)		Women (*n* = 121)		
	Mean or *n*	SD or %	Mean or *n*	SD or %	Mean or *n*	SD	
Age	20.39	1.31	20.36	1.43	20.41	1.20	
Race							
White, *n* (%)	140	63.3%	67	67.0%	73	60.3%	
Asian, *n* (%)	60	27.1%	26	26.0%	34	28.1%	
Black, *n* (%)	15	6.8%	4	4.0%	11	9.1%	
Other	6	2.7%	3	3.0%	3	2.5%	
Predominant ancestry^a^							
w1 > 0.9, *n* (%)	120	54.3%	59	59.0%	61	50.4%	
w2 > 0.9, *n* (%)	19	8.6%	11	11.0%	8	6.6%	
w3 > 0.9, *n* (%)	34	15.4%	14	14.0%	20	16.5%	
Other	47	21.3%	15	15.0%	32	26.4%	
Physiological measures							
Heart rate (per minute)^a^	68.10	10.85	66.64	10.94	69.31	10.67	
Systolic BP (mmHg)	113.06	13.93	118.46	13.65	108.60	12.56	^*^
Diastolic BP (mmHg)	63.41	8.62	63.71	9.19	63.17	8.16	
Respiratory rate (per minute)^b^	16.70	1.40	16.77	1.21	16.64	1.54	
Height (cm)	170.26	9.03	176.74	6.50	164.90	7.12	^*^
Weight (kg)	69.73	15.38	73.34	11.94	66.75	17.21	^*^
Body mass index (kg/m2)	24.01	4.82	23.44	3.33	24.48	5.73	
Trait measures							
NEO-PI-R neuroticism^b^	82.81	23.55	78.38	20.57	86.40	25.23	^*^
NEO-PI-R extraversion^b^	122.99	20.55	123.36	18.08	122.69	22.42	
NEO-PI-R openness^b^	122.33	18.72	119.61	18.14	124.54	18.97	
NEO-PI-R agreeableness^b^	121.70	20.34	115.74	18.36	126.53	20.64	^*^
NEO-PI-R conscientiousness^b^	122.91	20.11	120.77	18.31	124.64	21.38	
BIS-BAS behavioral inhibition^c^	19.79	3.63	18.89	3.65	20.52	3.46	^*^
BIS-BAS reward responsiveness^c^	18.14	1.83	18.02	1.93	18.23	1.75	
BIS-BAS drive^c^	11.47	2.68	11.49	2.68	11.44	2.69	
BIS-BAS fun seeking^c^	12.07	2.37	12.69	2.10	11.55	2.46	^*^
Appetitive Motivation Scale^d^	15.13	2.68	15.63	2.49	14.73	2.77	^*^
SPSRQ reward^e^	11.77	3.97	13.15	3.68	10.67	3.85	^*^
SPSRQ punishment^e^	10.30	4.97	9.71	4.91	10.77	4.99	
State measures							
PANAS positive^a^	29.17	7.27	29.83	7.19	28.63	7.32	
PANAS negative^a^	12.44	3.28	12.15	2.56	12.69	3.78	
PHQ-9^a^	3.34	3.74	2.85	2.63	3.75	4.43	
CESD^a^	8.76	7.83	7.80	5.63	9.56	9.22	
Perceived Stress Scale	11.65	6.16	10.55	5.32	12.58	6.67	
Beck Anxiety Inventory^c^	6.25	6.18	5.60	5.56	6.78	6.62	

NEO-PI-R, Neuroticism, Extraversion, Openness Personality Inventory—Revised;

BIS-BAS, Behavioral Inhibition and Approach Scales;

SPSRQ, Sensitivity to Punishment and Sensitivity to Reward Questionnaire;

PANAS, Positive and Negative Affect Schedule;

PHQ-9, Patient Health Questionnaire;

CESD, Center for Epidemiologic Studies Depression Scale

^*^: *P* < 0.05 Mann−Whitney test, male vs. female

a: *n* = 220, b: *n* = 219, c: *n* = 210, d: *n* = 208, e: *n* = 206

To adjust for multiple comparisons across seven reward-related questionnaire subscales, Bonferroni correction was applied (*P* = 0.05/7 = 0.0071). Similarly, adjustment was made for the four behavioral and SCR outcomes collected during the MID task (*P* = 0.05/4 = 0.0125), and for the five regions of interest in the fMRI experiment (*P* = 0.05/5 = 0.01). For whole-brain exploratory fMRI analyses in SPM, the false discovery rate was applied to adjust for multiple comparisons.

We performed a mediation analysis (‘mediation’ package, version 4.5.0) to evaluate whether the effects of sex on behavioral variables were statistically mediated by fMRI responses in our regions of interest. Significance of indirect effects was tested using bootstrapping. Unstandardized indirect effects and 95%-confidence intervals were calculated for 1000 bootstrapped samples.

## Results

### Participants

Demographics, physiological variables and psychological measures from the sample (121 women, 100 men) are shown in Tables 1, S1 and S2.

### Performance and ratings during the monetary incentive delay task

**Fig. 1:**
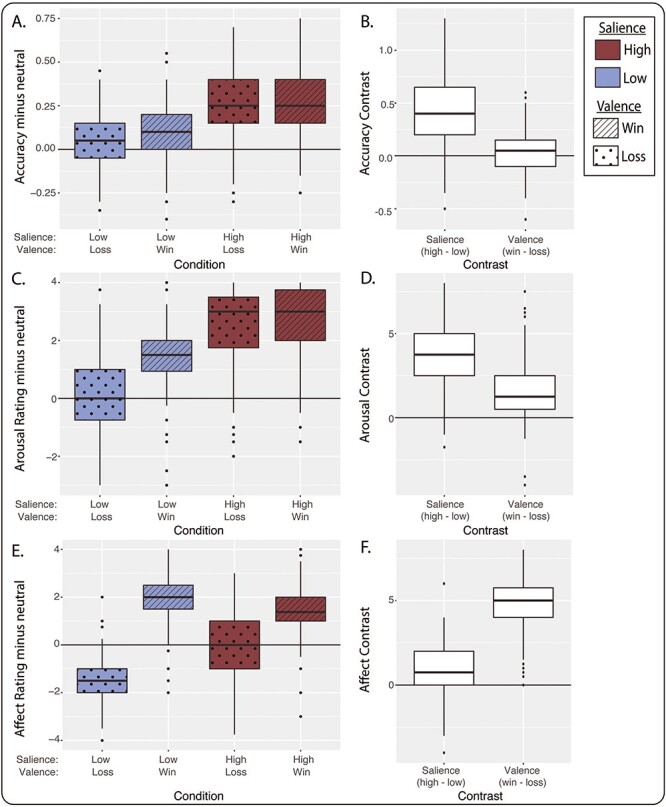
Behavior and ratings of stimuli during the MID task (*n* = 221). (A) The boxplots show task accuracy (i.e. hit rate) by condition across all subjects relative to accuracy for the neutral condition. (B) Accuracy data from A are replotted as salience contrast (high minus low) and valence contrast (win minus loss). (C) Arousal rating by condition, relative to neutral. (D) Salience and valence contrasts of arousal ratings. (E) Affect ratings by condition, relative to neutral. (F) Salience and valence contrasts of affect ratings. For all boxplots, center line is the median, box is interquartile range (IQR), whiskers are 1.5^*^IQR and the plotted points are outliers. High-salience conditions are shown in maroon and low-salience conditions are shown in blue. The salience contrast is calculated as high-salience conditions minus low-salience conditions, and the valence contrast is calculated as win conditions minus loss conditions.

Across the entire sample, accuracy (i.e. hit rate) was higher during high-salience conditions relative to low-salience conditions (Figure 1A, red vs blue boxes). This was expected because on high- but not low-salience trials, subjects could win or avoid losing money by performing more accurately. Accuracy was also modulated by valence: subjects were more accurate on win *vs* loss trials. The effects of task condition are shown most clearly by examining salience and valence contrasts (Figure 1B). A linear mixed model confirmed that accuracy depended on salience (*P* < 10^−15^, χ^2^ = 609, df = 1) and valence (*P* = 0.0055, χ^2^ = 4.76, df = 1), with a modest salience-by-valence interaction (*P* = 0.011, χ^2^ = 6.09, df = 1).

After performing the task, subjects rated their arousal (Figure 1C and D) and affect (Figure 1E and F) for each stimulus type on a 1-to-5 scale (see also Supplementary Figure S2). Like accuracy, self-reported arousal was greater for high-salience *vs* low-salience trials (*P* < 10^−15^, χ^2^ = 739, df = 1, linear mixed model) and for win *vs* loss trials (*P* < 10^−15^, χ^2^ = 84.9, df = 1, Figure 1C and D). A salience-by-valence interaction was also evident (*P* < 10^−15^, χ^2^ = 86.4, df = 1). Affect ratings showed a different pattern (Figure 1E and F). As expected, participants reported more positive affect on win trials than on loss trials (valence contrast, *P* < 10^−15^, χ^2^ = 1390, df = 1). Self-reported affect was also more positive when money was at stake (salience contrast, *P* < 10^−15^, χ^2^ = 22.9, df = 1) and a strong salience-by-valence interaction was observed (*P* < 10^−15^, χ^2^ = 343, df = 1).

### SCRs during the task

**Fig. 2:**
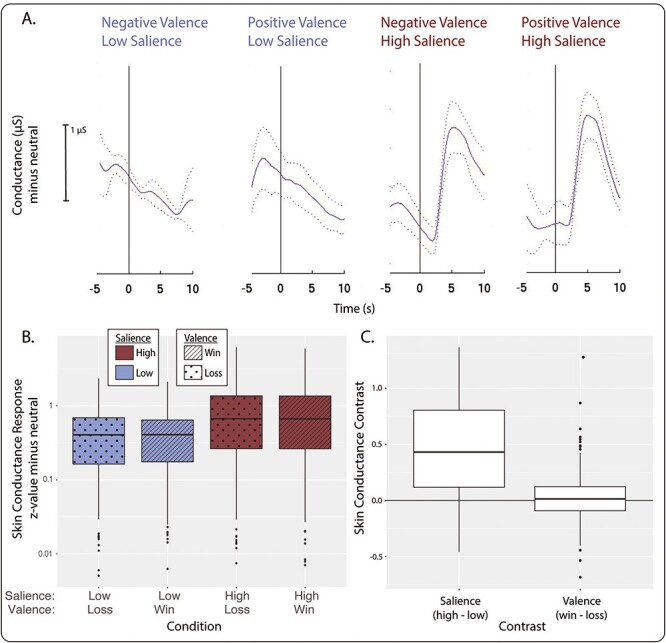
SCRs during the MID task (*n* = 201). (A) Example SCR by condition for one subject. Cue stimuli were presented at 0 s. The solid line is mean conductance relative to the neutral condition and the dotted lines represent +/− 1 standard error of the mean. (B) SCR z-values across all subjects. z-value calculated as mean peak-to-peak value minus the neutral condition divided by the standard deviation. (C) Salience and valence contrasts for the data shown in B. For all boxplots, center line is the median, box is interquartile range (IQR), whiskers are 1.5^*^IQR and the plotted points are outliers. The salience contrast is calculated as high-salience conditions minus low-salience conditions, and the valence contrast is calculated as win conditions minus loss conditions.

Skin conductance typically showed a biphasic response over the 10 s following stimulus presentation (Figure 2A). Stronger responses were observed under conditions of high salience relative to low salience (Figure 2B and C; salience contrast, *P* < 10^−15^, χ^2^ = 427, df = 1). Unlike behavioral measures, SCR amplitudes on win *vs* loss trials were equivalent (valence contrast, *P* = 0.41, χ^2^ = 1.08 df = 1), and no salience-by-valence interaction was detected (*P* = 0.50, χ^2^ = 0.0012, df = 1).

### Neural responses to monetary incentives

**Fig. 3:**
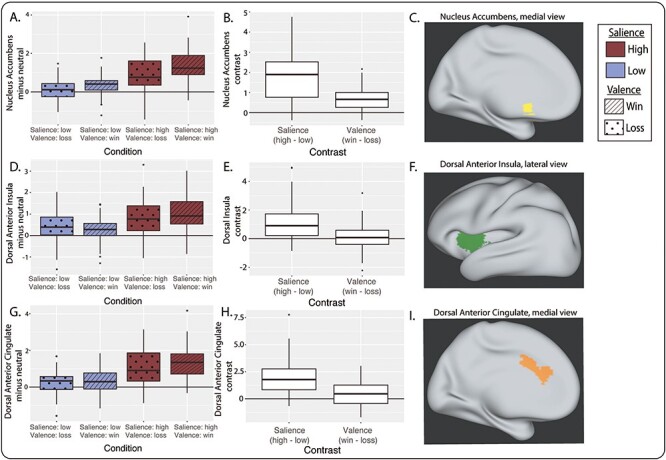
Neural activation during the MID task (*n* = 44). (A) Bilateral nucleus accumbens (NAc) BOLD contrast values by condition *vs* neutral. (B) Salience and valence BOLD contrasts for bilateral NAc response. (C) NAc region of interest in yellow on an inflated brain (medial view). (D) Bilateral dorsal AI BOLD contrast values by condition *vs* neutral. (E) Salience and valence BOLD contrasts for bilateral dorsal AI response. (F) Dorsal AI region of interest in green on an inflated brain (lateral view). (G) Bilateral dorsal anterior cingulate cortex (dACC) BOLD contrast values by condition *vs* neutral. (H) Salience and valence BOLD contrasts for bilateral dACC response. (I) dACC region of interest in orange on an inflated brain (medial view). For all boxplots, center line is the median, box is interquartile range (IQR), whiskers are 1.5^*^IQR and the plotted points are outliers. The salience BOLD contrast is calculated as high-salience conditions minus low-salience conditions, and the valence BOLD contrast is calculated as win conditions minus loss conditions.

As previously reported (Warthen *et al.,* 2018), the MID task activated the mesoaccumbal pathway. The NAc was more responsive to high *vs* low salience stimuli (*P* < 10^−15^, χ^2^ = 135, df = 1) and win *vs* loss stimuli (*P* = 0.0016, χ^2^ = 9.99, df = 1; Figure 3A and B). The midbrain was responsive to salience (*P* < 10^−15^, χ^2^ = 120, df = 1) with no significant valence effect (*P* = 0.23, χ^2^ = 1.47, df = 1). No salience-by-valence interaction was exhibited in either region (*P* > 0.05).

This task also activated the principal nodes of the salience network: the dACC and bilateral AI. Unlike the NAc, the AI was primarily sensitive to stimulus salience (dorsal AI: *P* = 7.5 × 10^−12^, χ^2^ = 46.9, df = 1; ventral AI: *P* = 2.6 × 10^−9^, χ^2^ = 35.5, df = 1) and not valence (dorsal: *P* = 0.64, χ^2^ = 0.21, df = 1; ventral: *P* = 0.31, χ^2^ = 1.02, df = 1; Figure 3). A modest salience-by-valence interaction was detected in the AI (dorsal: *P* = 0.023, χ^2^ = 5.19, df = 1; ventral: *P* = 0.056, χ^2^ = 3.64, df = 1). Similar to the AI, dACC activity was modulated by salience (*P* < 10^−15^, χ^2^ = 99.7, df = 1), with no effect of valence (*P* = 0.17, χ^2^ = 1.85, df = 1) or salience-by-valence interaction (*P* = 0.29, χ^2^ = 1.12, df = 1).

### Sex differences in reward-related traits

We found significant sex differences in three reward-related trait questionnaires (Table 1). On the BIS-BAS scale, women reported greater Behavioral Inhibition (Hedges’ *g* = 0.46, *P* = 0.0027, linear model), whereas men reported greater Fun Seeking (*g* = 0.49, *P* = 7.4 × 10^−4^). No significant differences were found for the BIS-BAS Reward Responsiveness or Drive subscales (*g* = 0.12, *P* = 0.53; *g* = 0.019, *P* = 0.91, respectively). Scores on the SPSRQ Reward subscale were greater in men than women (*g* = 0.61, *P* = 1.0 × 10^−5^), but no sex differences were found for the Punishment subscale (*g* = 0.24, *P* = 0.14). Men scored higher than women on the Appetitive Motivation Scale (*g* = 0.34, *P* = 0.018). Sex differences in the BIS-BAS Behavioral Inhibition, BIS-BAS Fun Seeking and SPSRQ Reward subscales remained significant after Bonferroni correction for testing seven reward-related questionnaire subscales (uncorrected *P* < 0.0071). Consistent with previous reports (Costa and McCrae, 1992; Shen *et al.,* 2017), we also found significant differences between men and women in Neuroticism, Agreeableness, height, weight and systolic blood pressure (Table 1).

### Sex differences in performance and subjective ratings

**Fig. 4:**
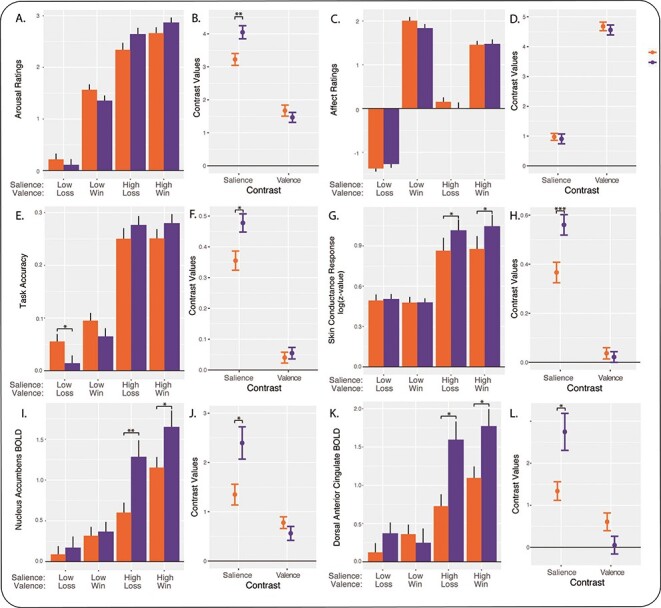
Summary of sex differences during the MID task. Women are shown in orange and men are shown in purple. (A) Arousal ratings by task condition (mean +/− standard error of the mean, relative to the neutral condition). (B) Data from A are shown as salience and valence contrasts of arousal rating (mean +/− standard error of the mean). (C) Affect ratings by condition, relative to neutral. (D) Salience and valence contrasts of affect ratings. (E) Accuracy by condition, relative to neutral. (F) Salience and valence contrasts of accuracy. (G) SCR amplitude (z-value) by condition, relative to neutral. (H) Salience and valence contrasts of SCR. (I) NAc response by condition, relative to neutral. (J) Salience and valence contrasts of NAc response. (K) dACC response by condition, relative to neutral. (L) Salience and valence contrasts of dACC response. ^*^P < 0.05, ^*^^*^P < 0.005, ^*^^*^^*^P < 0.001, Mann−Whitney test (not adjusted for NPY group). The salience contrast is calculated as high-salience conditions minus low-salience conditions, and the valence contrast is calculated as win conditions minus loss conditions.

Linear-mixed-model analysis of accuracy demonstrated a significant sex-by-salience interaction (*P* = 0.00012) and no main effect of sex or sex-by-valence interaction (*P* > 0.05; Supplementary Table S5). Average accuracy across task conditions was similar between men and women (*g* = 0.022, *P* = 0.74, linear model). However, accuracy of males depended more strongly on salience than did the accuracy of females (*g* = 0.39, *P* = 0.0041; salience contrast, Figure 4E and F). Men also earned more money during the task (*g* = 0.31, *P* = 0.023) due to their higher accuracy on high-salience trials. No sex differences were found for accuracy on win *vs* loss trials (*g* = 0.073, *P* = 0.59; valence contrast, Figure 4E and F). No sex differences were evident for the neutral condition (*g* = 0.066, *P* = 0.66).

The pattern of sex differences for arousal ratings was similar to the pattern for accuracy. Linear-mixed-model analysis revealed a significant sex-by-salience interaction (*P* = 0.00017) and no main effect of sex or sex-by-valence interaction (*P* > 0.05; Supplementary Table S5). Men reported greater differences in subjective arousal for high-salience *vs* low-salience stimuli (*g* = 0.42, *P* = 0.0022; salience contrast, Figure 4A and B). The valence contrast of arousal ratings did not differ between men and women (*g* = 0.13, *P* = 0.37), indicating that the difference in subjective arousal for win *vs* loss was similar for the two sexes. No difference between sexes was found for the neutral condition (*g* = 0.11, *P* = 0.45).

Unlike arousal ratings, linear-mixed-model analysis of affect ratings showed no significant main effect of sex, sex-by-salience interaction or sex-by-valence interaction (*P* > 0.05; Supplementary Table S5). We found no sex differences for the salience contrast (*g* = 0.046, *P* = 0.73) or valence contrast (*g* = 0.074, *P* = 0.58; Figure 4C and D). This indicated that men and women experienced similar differences in subjective affect between high- and low-salience trials, and between win and loss trials. Affect ratings for the neutral condition showed no sex differences (*g* = 0.033, *P* = 0.81).

### Sex differences in autonomic responses

Linear-mixed-model analysis of SCR revealed a significant sex-by-salience interaction (*P* = 0.0000082) and no main effect of sex or sex-by-valence interaction (*P* > 0.05; Supplementary Table S5). SCR for high- *vs* low-salience stimuli was greater among male participants (salience contrast, *g* = 0.46, *P* = 0.0013, linear model; Figure 4G and H). No sex differences were found for SCR to win *vs* loss stimuli (valence contrast, *g* = 0.067, *P* = 0.64). Thus, sex differences observed in SCR mirrored those observed for behavioral accuracy and subjective arousal. Furthermore, the sex difference detected in SCR salience contrast remained significant while controlling for accuracy (*P* = 0.016) or arousal ratings (*P* = 0.043), suggesting that sex differences in autonomic arousal are not simply explained by differences in behavior.

### Sex differences in neural responses

A linear mixed model was applied to each of five regions of interest. We found a significant sex-by-salience interaction in all regions (*P* < 2 × 10^−12^), and a main effect of sex for all regions of interest except the midbrain (*P* < 0.05). In addition, a sex-by-valence interaction was detected in the NAc and dACC (*P* < 0.05; Supplementary Table S5).

Compared to women, men showed greater sensitivity to stimulus salience within the mesoaccumbal pathway and salience network. Responses to high- *vs* low-salience stimuli were greater in men than in women in the NAc (salience contrast, *g* = 0.84, *P* = 0.0050, linear model; Figure 4I and J). A similar sex difference was found in the midbrain (*g* = 0.61, *P* = 0.049). We also found sex differences in the cortical nodes of the salience network. Compared to women, salience contrasts were greater among men in the dACC (*g* = 0.89, *P* = 0.0050; Figure 4K and L), dorsal AI (*g* = 0.76, *P* = 0.016) and ventral AI (*g* = 0.73, *P* = 0.018). Unlike findings for the salience contrast, no significant sex differences were found for the valence contrast (win *vs* loss) in any of these regions of interest (*g* = 0.16 to 0.55, *P* = 0.08 to 0.60). No sex differences were found in other brain regions using whole-brain correction for multiple comparisons (Supplementary Table S4).

### Multi-level analyses

Because the behavioral, autonomic and neural outcomes we measured were intercorrelated (Supplementary Figure S3), we evaluated sex differences using linear mixed models in which level of analysis was added as a fixed-effect predictor. For the fMRI subsample (*n* = 44), five levels (regions of interest) were modeled. For the larger sample (*n* = 201), four levels were modeled (accuracy, arousal rating, affect rating and SCR). Analysis of salience contrasts confirmed a significant effect of sex for behavioral and autonomic responses (χ^2^ = 10.7, df = 1, *P* = 0.0011) and for neural responses (χ^2^ = 8.3, df = 1, *P* = 0.0039). Similar models of valence contrasts revealed no effect of sex (*P* > 0.05). These findings confirmed that these sex differences in responsiveness to salience remained after accounting for comparison of multiple correlated outcomes within and across levels of analysis.

### Mediation analyses

In a set of post hoc, exploratory, statistical mediation analyses, we determined whether neural responses (BOLD salience contrast) mediated sex effects on behavioral and autonomic responses (salience contrasts of arousal ratings, task accuracy and SCR). Overall, we found that each node of the mesoaccumbal pathway and salience network was able to fully mediate the effect of sex on accuracy and arousal ratings (Supplementary Results and Supplementary Figure S5).

### Control analyses

There was no relationship between sex and NPY group (*P* = 0.84, linear model), race (*P* = 0.61, linear model) or ancestry (*P* = 0.18, linear model) in our subjects. Women and men in our sample differed with respect to physiological measures (systolic blood pressure, height and weight), personality traits (neuroticism and agreeableness) and clinical diagnoses (social phobia and generalized anxiety disorder). However, control analyses indicated that these potential confounding variables did not account for the behavioral and physiological sex differences we found in the reward system (see Supplementary Results).

## Discussion

This study revealed differences in behavioral, autonomic and neural responses between men and women during motivated behavior. We found that men were more sensitive to the behavioral relevance (salience) of incentive stimuli, and that women and men responded similarly with respect to stimulus valence (i.e. win *vs* loss of money). Importantly, these sex differences were observed consistently across neurobehavioral levels, which increases confidence in our findings. Neural differences between women and men were evident in the mesoaccumbal pathway and salience network, and effects on behavior were mediated by the BOLD signal from regions of interest in these pathways. To our knowledge, this is the first report of robust sex differences in the reward system across multiple behavioral and physiological levels of analysis.

The finding of sex differences in sensitivity to salience, but not valence, indicates that the behavioral and neural responsiveness of men and women differed when presented with a behaviorally relevant cue and, furthermore, that the responses of women and men to win *vs* loss (reward *vs* punishment) were not different. Given the greater propensity of women to develop mood and anxiety disorders, we had hypothesized that women might respond more strongly to negative-valence stimuli than to positive-valence stimuli, but this was not the case. If risk for these disorders does in fact originate from a difference in reward and salience processing, the elevated risk among women may be due to a lower response to behaviorally relevant stimuli in general, rather than hyperresponsiveness to negative stimuli. The additional finding that each node of the mesoaccumbal pathway and salience network was able to fully mediate the effects of sex on arousal ratings and task performance suggests that the average differences in behavior that we observed between women and men are underpinned by fundamental differences in neural processing between the sexes.

Our findings are comparable to several previous neuroimaging studies that tested sex differences in mesoaccumbal function. An early positron emission tomography study reported greater amphetamine-induced dopamine release in the NAc among men relative to women (Munro *et al.,* 2006) and similar findings were reported for response to nicotine (Cosgrove *et al.,* 2014). Several early fMRI studies reported no significant sex differences in the NAc or midbrain during anticipation or receipt of monetary rewards (Dreher *et al.,* 2007; Spreckelmeyer *et al.,* 2009; Diekhof *et al.,* 2012). Although task differences might explain why our findings differ from those previous studies, the apparent discrepancy may be due to low power in those studies. In a more recent fMRI study, NAc responses to visual food cues were found to be greater in women than in men while fasting (Legget *et al.,* 2018). Two recent, well-powered, fMRI studies reported greater NAc responses in males relative to females using monetary tasks that differ from ours. Using an event-related gambling task, Alarcón *et al.* (2017) demonstrated greater response of right NAc (but not left NAc) to receipt of monetary rewards among male adolescents. Curtis *et al.* (2019) analyzed Human Connectome Project data acquired with a block-design guessing task, and found greater bilateral NAc activation among men both during predominant-win blocks and during predominant-loss blocks.

Our results are consistent with the latter two reports, but we build upon those studies in important ways. First, we examined reward system function across multiple levels of analysis—psychological traits, task performance, subjective ratings, autonomic responses and neural activity—and demonstrated convergent evidence of sex differences. Second, we employed a well-established task known to engage the mesoaccumbal pathway and salience network during anticipation of monetary incentives and revealed sex differences with large effect sizes (*g* = 0.8 to 1.7) in the NAc, AI and dACC. Third, we directly compared responses to positive- *vs* negative-valence stimuli (i.e. monetary gain *vs* loss) to show that sex differences are not specific to rewards but rather apply to salient stimuli more generally.

The sex differences we found in the salience network differ in some ways from the findings of previous studies. The study by Curtis *et al.* (2019), which used a guessing task with a block-design, reported that activity in the insula was greater in men than in women under the predominant-win condition, similar to our results; but our findings appear to disagree with theirs for the predominant-loss condition and for the anterior cingulate cortex (Curtis *et al.,* 2019). Morgan *et al.* (2013) used a similar task in a study of adolescents and found that anterior cingulate responses during reward anticipation were greater among girls—opposite in direction to our results. Similarly, our findings seem discrepant with the study by Legget *et al.* (2018), which found that insula responses to visual food cues were greater in women than in men seems unnecessary. However, these apparent discrepancies are not surprising given the substantial differences in fMRI tasks used. For example, our subjects had to attend closely to visual cues in order to perform well, which was not the case for the other tasks. Interestingly, a recent study found sex-specific effects of cortisol administration on anterior cingulate responses to anticipation of verbal but not monetary rewards, suggesting that the type of incentive and the hormonal context may be important moderators of sex differences in anterior cingulate function (Kinner *et al.,* 2016). Disentangling these sex differences will require the use of task designs that distinguish the valence of incentives (e.g. winning *vs* avoiding loss) and different phases of motivated behavior (e.g. anticipation *vs* consummation *vs* learning).

The human sex differences we found diverge in some ways from previous findings with rodents. For example, in a recently reported series of behavioral studies comparing female and male rats, females more rapidly learned to avoid punishment and were more sensitive to risk of punishment during reward-seeking behavior (Chowdhury *et al.,* 2019). If similar sex differences were present in humans, then one might expect to observe greater responsiveness of females to loss *vs* win in our experiments, but instead we found no behavioral or physiological sex differences in responses by valence. Previous behavioral experiments using rat addiction models have typically found more rapid acquisition and escalation of drug self-administration, as well as stronger motivational withdrawal and reinstatement behaviors, among females (Becker, 2016; Becker and Koob, 2016). Similarly, a study of neural activation (Fos expression) during cue-induced reinstatement of cocaine self-administration reported greater activation among females in brain regions including the NAc, ventral tegmental area and agranular insula (homologous to AI) (Zhou *et al.,* 2014). On the face of it, this greater behavioral and neural responsiveness of female rats appears opposite to the direction of sex differences we found in humans. On the other hand, some rodent results seem more in line with our findings. For example, male rats were reported to have higher dopamine concentrations in NAc (Cummings *et al.,* 2014) and males had higher expression of striatal D1 dopamine receptors, which are excitatory (Becker, 2016). Furthermore, males typically show greater stimulant-induced dopamine release in the NAc and dorsal striatum—effects which may be influenced by estrogen and progesterone (Castner *et al.,* 1993; Larson *et al.,* 2007; Cummings *et al.,* 2014; Gillies *et al.,* 2014). The apparent discrepancies of our findings *vs* rodent findings, and discrepancies between rodent experiments, may arise from differences in behavioral paradigms (i.e. money *vs* food or drugs), or from inherent species differences.

Our study has several important limitations. First, the age range of our subjects was limited to 18–22 years in order to minimize age-related variability, so it remains unknown whether our findings generalize to other ages. Future studies of other age groups are warranted to determine how these sex differences depend on developmental stage. Second, we used a behavioral task that focused on the anticipatory or preparatory phase of motivated behavior, and that utilized gain or loss of money as the incentive, not varying in incentive levels. While this task has certain advantages—it is well validated and produces robust neural responses—the sex differences we found may not generalize to other phases of motivated behavior (e.g. receipt or consummation) or other kinds of incentives (e.g. social feedback, food or drugs). Indeed, there is some evidence that NAc responses to visual food stimuli may be greater in women than in men (Legget *et al.,* 2018). Third (and related to the previous point), the task we used activated a limited set of brain regions, so it may not have allowed us to detect sex differences outside of these regions. Fourth, we did not directly measure menstrual cycle or hormonal milieu, which may impact reward function (Terner and de Wit, 2006; Allen *et al.,* 2010; Fattore *et al.,* 2014), and did not directly assess gender identity. Additionally, our imaging results should be replicated in a larger sample size.

The differences in neurobehavioral function we found between females and males may have clinical implications. The mediation of sex effects on behavior by region of interest indicates that sex is not only exerting effects on brain activity and behavior, but that these effects are linked. We found that psychological and physiological arousal was greater in men than in women when presented with high-salience *vs* low-salience stimuli. This predisposition of men may contribute to their greater likelihood to initially participate in addictive behaviors such as gambling or drug use (Fattore *et al.,* 2014; Becker, 2016; Riley *et al.,* 2018; Mayo *et al.,* 2019). Furthermore, if women initially experience less arousal but the potential range of psychological and physiological arousal is similar to that of men, then this phenotype might facilitate more rapid escalation of addictive behaviors in women (Fattore *et al.,* 2014; Becker, 2016; Riley *et al.,* 2018). Decreased mesoaccumbal function and electrodermal hyporeactivity have previously been linked with depression and suicide (Thorell *et al.,* 2013; Zhang *et al.,* 2013; Zhang *et al.,* 2016), so the lower activation of the mesoaccumbal pathway and lower SCRs we found among a community sample of women compared to men may also contribute to the higher risk of incident depression among women.

The sex differences we found in this study are likely caused by multiple factors. Females and males are genetically different, but over the course of development they are also exposed to divergent hormonal environments and distinct social influences. The psychological and physiological differences we found in reward function probably arise from a combination of genetic factors and sex-specific socialization. Brain and behavior differences between men and women provide insight into mechanisms of reward function and dysfunction. However, it is important to remember that the distributions of female and male groups overlap substantially, and sex is just one of many individual characteristics that contribute to human variation. The sex differences we describe here should be considered population-level, not individual-level, phenomena.

## Supplementary Material

scan-20-007-File007_nsaa104

scan-20-007-File008_nsaa104

scan-20-007-File009_nsaa104

scan-20-007-File010_nsaa104

scan-20-007-File011_nsaa104

scan-20-007-File012_nsaa104

scan-20-007-File013_nsaa104

scan-20-007-File014_nsaa104

scan-20-007-File015_nsaa104

scan-20-007-File016_nsaa104

scan-20-007-File017_nsaa104
