# Corrigendum to: Sex differences in the human reward system: convergent behavioral, autonomic and neural evidence

**DOI:** 10.1093/scan/nsab051

**Published:** 2021-05-07

**Authors:** Katherine G Warthen, Alita Boyse-Peacor, Keith G Jones, Benjamin Sanford, Tiffany M Love, Brian J Mickey

**Affiliations:** Department of Psychiatry, University of Utah, Salt Lake City, UT 84108, USA; College of Arts and Sciences, Oberlin College, Oberlin, OH 44074, USA; Department of Psychiatry, University of Utah, Salt Lake City, UT 84108, USA; Department of Psychiatry, University of Michigan, Ann Arbor, MI 48109, USA; Department of Psychiatry, University of Utah, Salt Lake City, UT 84108, USA; Department of Psychiatry, University of Utah, Salt Lake City, UT 84108, USA; Department of Psychiatry, University of Michigan, Ann Arbor, MI 48109, USA

In the originally published version of this manuscript, there were errors in the description of effect sizes in the Abstract and Discussion sections.

In the Abstract, there are two sentences that incorrectly state the range of effect sizes: In the sentence “In a subsample studied with functional magnetic resonance imaging (*n* = 44), men exhibited greater responsiveness to stimulus salience in the nucleus accumbens, midbrain, anterior insula and dorsal anterior cingulate (*P* < 0.02, *g* = 0.86 to 1.7).”, the *g* range should be *g* = 0.73 to 0.89.

In the fourth paragraph in the Discussion: In the sentence ‘Second, we employed a well-established task known to engage the mesoaccumbal pathway and salience network during anticipation of monetary incentives and revealed sex differences with large effect sizes (*g* = 0.8 to 1.7) in the NAc, AI and dACC.’, the *g* range should be *g* = 0.73 to 0.89.

These errors have been corrected online.

