# Supplementary material for: Sex differences in the human reward system: convergent behavioral, autonomic and neural evidence
Source: Soc Cogn Affect Neurosci. 2020 Jul 30;15(7):789–801. doi: 10.1093/scan/nsaa104 (PMC7511890; doi:10.1093/scan/nsaa104)
Supplement: scan-20-007-File007_nsaa104 [file scan-20-007-file007_nsaa104.docx]

**­Supplemental Methods**

*Skin conductance responses*

Skin conductance (electrodermal activity) was measured from the palmar surface of the left (non-dominant) hand using a Biopac system (MP150; Goleta, CA) with a sampling rate of 1 kHz. Electrodes were placed over the thenar and hypothenar eminences. In a few cases when initial recordings were of low quality, electrodes were instead placed on the index and middle fingers. Recordings with a mean skin conductance level of less than 1 μS across an entire run, and recordings in which the mean conductance was more than 2-fold lower in one run compared to the other run, were considered low quality and were eliminated from analysis. SCR for each of the 5 task conditions was recorded with 20 repetitions (pseudo-random order, 10 repetitions per run, 2 runs). Skin conductance level during the period 5 s before and 10 s after each cue presentation was extracted, and the SCR for that trial was calculated by subtracting the mean conductance value over the 15-second epoch. A semi-automated procedure was used to identify and remove trials with obvious artifacts (<1% of trials). The average SCR for each condition (high-salience win, high-salience loss, low-salience win, low-salience loss) was a time-varying function, computed as the mean response across 20 trials of that condition minus the mean response across 20 neutral trials (Figure 2A). The peak-to-peak value of the SCR for each condition was calculated as the difference between the minimum value and the maximum value of the average SCR during the 10 seconds after cue presentation. SCR z-scores were calculated as the peak-to-peak value divided by the pooled standard error. SCR z-scores were then log10 transformed for statistical analyses.

*Task Details*

The monetary incentive delay (MID) task was implemented with E-Prime 2.0 software (Psychology Software Tools, RRID:SCR_009567). Participants first performed the task outside the MRI scanner during the Phase 1 visit and later performed the task inside the scanner during Phase 2. Subjects could earn additional money depending on task performance ($0-10 during Phase 1 and $0-20 during Phase 2). To enhance motivation, participants were shown the money (US dollar bills) they could earn before starting the task.

As described in Materials and Methods, the five possible trial types were: *high* salience and *positive* valence (HP); *high* salience and *negative* valence (HN); *low* salience and *positive* valence (LP); *low* salience and *negative* valence (LN); and neutral. On high-salience trials, participants had the potential to gain or avoid losing $1 if they hit the target. On low-salience trials, participants gained or lost $1 regardless of performance. (In other words, the outcome was delivered with the cue.) On neutral trials, no money was at stake. At the onset of each trial, the cue was presented on screen for 2 s, followed by the appearance of a crosshairs for a variable delay interval (1.3 to 1.8 s), followed by a target (a solid triangle) for a brief interval of ~200 ms. Participants were instructed to hit the target by pressing a response button using the right index finger before the target disappeared at the end of the target interval. The target interval was determined adaptively on each trial, based on the participant's performance on recent non-neutral trials, such that the average hit rate would approximate 67%. Feedback about performance on that trial was then shown on the screen for a variable interval (1.5 to 7.5 s) before the beginning of the next trial. Each subject completed two 8.5-minute MRI task sessions. During each session, each of the five trial types was repeated 10 times for a total of 50 trials. Each block of five trials was delivered in pseudorandom order (the same order for all participants). Reaction time and hit/miss were recorded on each trial. The primary performance outcome, accuracy (i.e., hit rate), was subsequently calculated per subject per trial type. Due to software limitations, reaction times were not recorded on miss trials, thus longer reaction times were missing. Each subject's reaction time for each trial type was therefore calculated as the first quartile of reaction times for those trials.

Participants also rated *Arousal* and *Affect* for each cue on a 5-point scale at the end of the Phase 1 task session. For affect, participants rated "how positive or negative you feel," from 1 ("negative") to 5 ("positive"). Arousal was rated based on "how aroused you feel," from 1 ("not aroused") to 5 ("very aroused”). Each cue was presented 4 times in pseudorandom order and the rating was calculated as the average of the 4 responses.

*Imaging*

T2* weighted images (TR=2s, TE=28ms, flip angle=90°, 39 transverse slices, slice thickness=3.5mm, slice gap=0mm, FOV=64x64 matrix, 3.75x3.75mm) were collected with a 15-channel head coil using single-shot echo-planar imaging. Two-hundred and fifty volumes were acquired over 8.5 minutes during the fMRI task. The first 5 volumes of each session were discarded for image stabilization and slice-time correction was applied using SPM with the middle slice as reference. Two-pass rigid-body least-squares motion correction was used, registering images to the first image of the first run, and then registering images to the mean of the first pass. Images were interpolated with a 4^th^ degree B-spline. The T1-weighted high-resolution image (turbo-field-echo, TR=9.8ms, TE=4.6ms, flip angle=8°, 1x1x1mm voxel size) was co-registered to the mean functional image with a rigid-body transform using a normalized mutual-information cost function. The registered T1-weighted image was segmented into gray matter, white matter, and cerebrospinal fluid images with the VBM8 toolbox in SPM. DARTEL was used to normalize the gray and white matter images to MNI space using the MNI-space template provided by the VBM8 toolbox. The DARTEL estimated warp was then applied to the motion-corrected functional images, which were then resliced to 3x3x3mm voxels. Smoothing was then performed with an 8-mm FWHM isotropic kernel.

SPM8 was used to perform first-level modeling using univariate linear regression, applying generalized least squares with a global AR(1) autocorrelation model and a high-pass filter with a 128-s cutoff. The cue on each trial of the MID task was modeled with an event-related design. The independent variables in the model included task regressors (onset of the 5 task cue types) convolved with the canonical hemodynamic response function, realignment parameters from motion correction along with their first derivatives, quadratic terms for the original and derivatives (24 parameters per run), as well as the top five principal components estimated from CSF masks for each subject (Behzadi *et al.*, 2007).

*Brain regions of Interest­­*
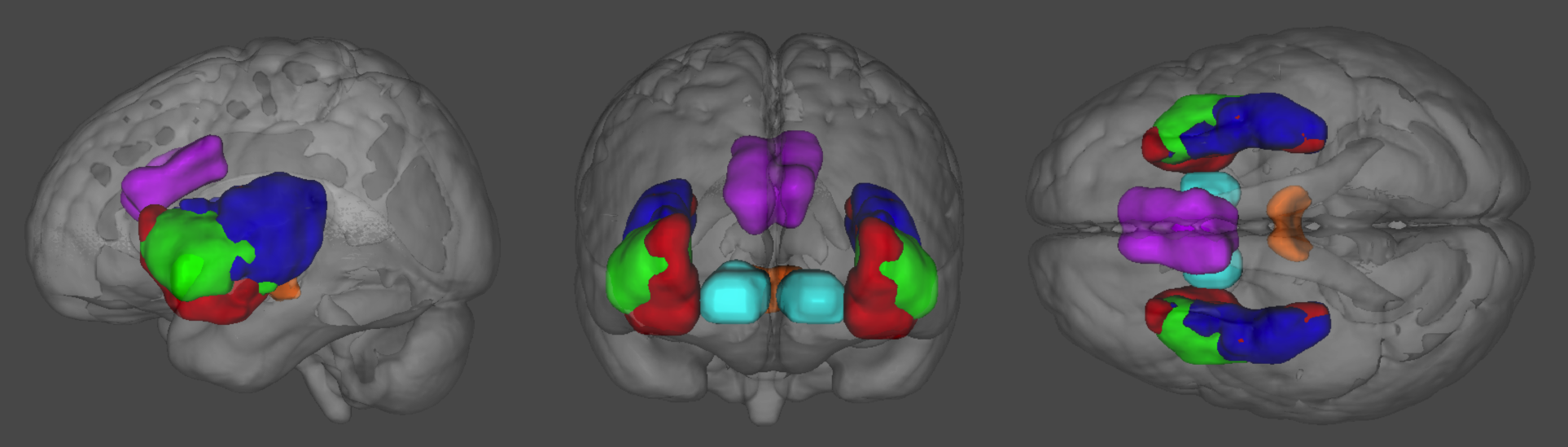


Figure S1, Left lateral, frontal, and inferior views showing regions of interest. Light blue is the nucleus accumbens (Warthen et al., 2018), red is the ventral anterior insula, green is the dorsal anterior insula (Chang et al., 2013), purple is the dorsal anterior cingulate cortex (Desikan et al., 2006), and orange is the midbrain region of interest (Mickey et al., 2016). The posterior insula (dark blue (Chang et al., 2013)) is shown for completeness but was not a region of interest.

*Genotyping*

Functional MRI data were collected for subjects classified into either of two neuropeptide Y genotype groups, as previously described (Warthen *et al.*, 2018). All fMRI analyses here include genotype group as a covariate of no interest. DNA was isolated from whole blood using a Gentra Puregene Blood Kit (Qiagen, Redwood City, CA). Sex and ancestry were estimated from genome-wide data (PsychArray, version 1.0, Illumina) using ADMIXTURE (version 1.3.0)(Alexander et al, 2009) with PLINK (version 1.07)(Purcell et al, 2007), as previously described (Warthen *et al.*, 2018).

**Supplemental Results**

*Participants*

Table S1 compares demographics, genetically estimated ancestry, physiological measures, and questionnaire results for the 44 subjects with usable MRI data versus subjects who took part in the MID task but were not imaged. On average, imaged subjects had nominally higher heart rate (p=0.037), higher diastolic blood pressure (p=0.045), lower NEO-PI-R Extraversion (p=0.010), lower BIS-BAS Reward Responsiveness (p=0.045), lower BIS-BAS Fun Seeking (p=0.039), and lower Appetitive Motivation Scale score (p=0.0086) (Table S1). None of these differences remained significant after correcting for 29 comparisons (all p>0.10, false-discovery-rate adjustment). Out of the total 221 subjects, two were taking hormonal birth control. None of the final 44 imaged subjects were on any form of birth control. We conclude that the imaged subjects were similar to the remainder of the sample on demographic, physiological, and clinical variables.

Although subjects self-reported as healthy, a small percentage of people met MINI criteria for a current psychiatric disorder (Table S2). These subjects were included in the study in an attempt to avoid creating a "super-normal" sample. Additional psychiatric interview diagnoses and ancestry information for all subjects are shown in Table S2. Table S3 shows a comparison of demographics, trait, and state information between imaged men and women.

*Subjective ratings of cue stimuli*

Figure S2 shows individual ratings per task condition across the dimensions of arousal and affect. Ratings of the neutral condition (grey) were, as expected, neither particularly negative or positive, with a medium to low arousal rating. For high salience conditions (blue and red), arousal ratings were generally high as predicted. However, affect ratings for high-salience losses were surprisingly variable, ranging from very negative to very positive. For win conditions, whether low or high salience, affect ratings were almost uniformly positive as expected. Low salience conditions were potentially more interesting when differentiating between win and loss conditions, where wins are rated as more arousing than losses.


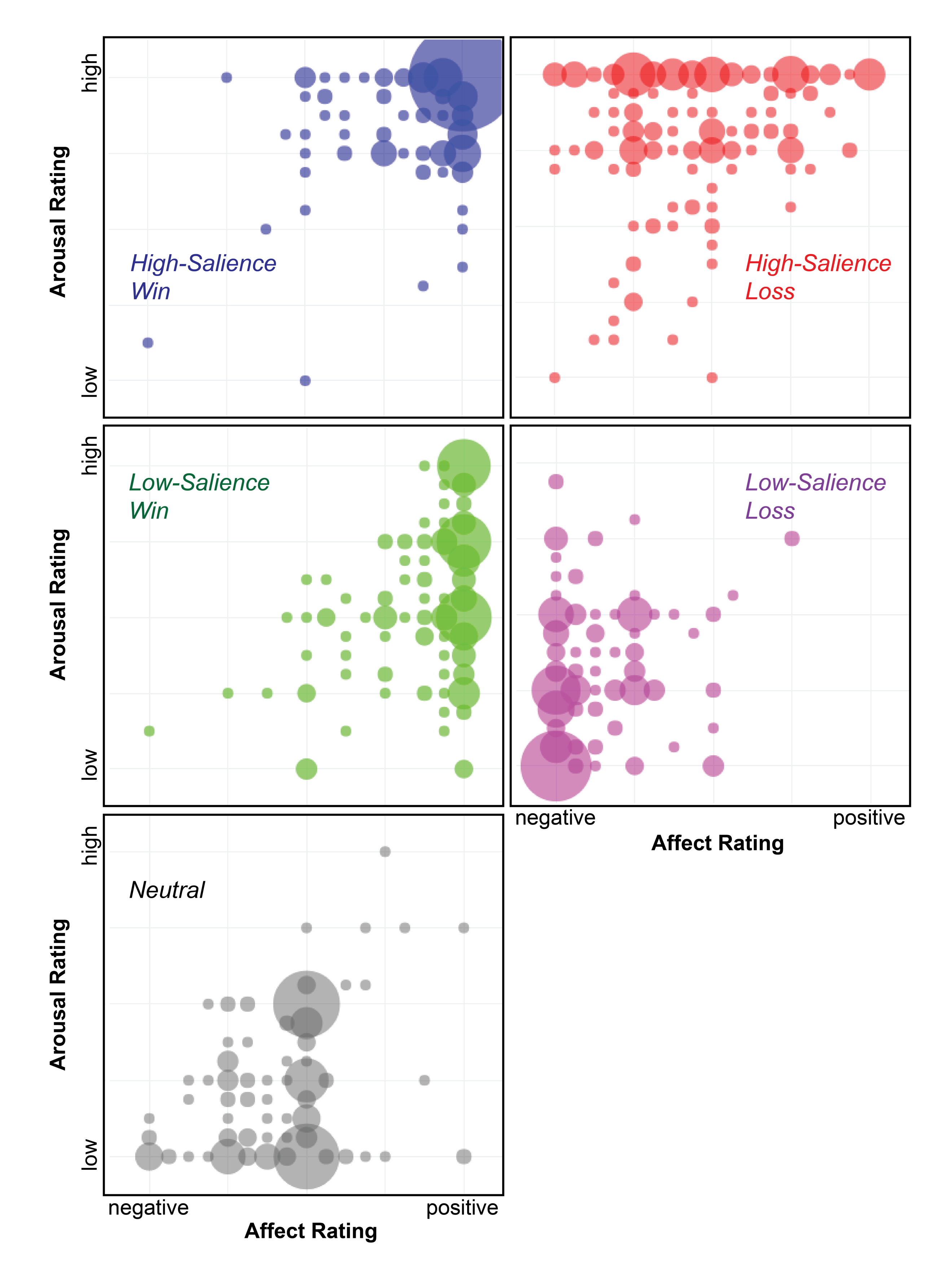


Figure S2, Arousal and affect ratings (range 1-5) by task condition. Sizes of circles represent number of subjects that provided that rating.

*Associations between physiological measurements and behavior*

Correlation analyses showed modest associations between most reward-related traits, performance, stimulus ratings, SCR, and neural responses (Figure S3). The highest inter-correlations were found among neural regions of interest.

Figure S3, Spearman correlation matrices for physiological, behavioral, trait, and state measures. *p < 0.05, **p < 0.01, ***p < 0.001, confidence level=0.95.

*Task measures in imaged subjects*

The subset of imaged subjects only (n=44) displayed similar sex differences outcomes to the entire cohort in measures of arousal rating salience contrast (p=0.026, linear model), task accuracy (p=0.033, linear model), and the lack of sex differences in valence rating salience contrast (p=0.41, linear model). Sex difference in SCR salience contrast in the 40 subjects with imaging data and skin conductance data passing quality control threshold (see methods) did not reach statistical significance (p=0.14, linear model). Among the 44 imaged subjects the sex difference in ventral anterior insula salience contrast were diminished (lost statistical significance) with the addition of the arousal rating salience contrast as an additional covariate in the linear model (p=0.13) or accuracy salience contrast (p=0.088), but remained with the addition affect rating as a covariate (p=0.016). Similar results were found for the dorsal anterior insula (covariate: arousal rating p=0.090, affect rating p=0.014, accuracy p=0.066), NAc (covariate: arousal rating p=0.061, affect rating p=0.0054, accuracy p=0.091), dACC (covariate: arousal rating p=0.031, affect rating p=0.0031, accuracy p=0.029), and midbrain (covariate: arousal rating p=0.28, affect rating p=0.029, accuracy p=0.22). The dACC was the only region where the sex effect remained significant even with the addition of behavioral covariates in this smaller population.

*Control analyses*

As shown in Tables 1 and S2, the women and men in our sample differed with respect to physiological measures (systolic blood pressure, height, and weight), personality traits (neuroticism and agreeableness), and clinical diagnoses (social phobia and generalized anxiety disorder), raising the possibility that these confounding variables were responsible for the behavioral and physiological sex differences we found. To evaluate that possibility, we tested for associations of each of those variables with salience contrasts of task performance, stimulus ratings, SCR, and neural responses in regions of interest. We found no significant associations (p>0.05, linear model), with the exception of height being positively associated with SCR (p=0.009). However, height did not predict SCR when men and women were analyzed separately (men: p=0.16, women: p=0.30). We conclude that these physiological and clinical variables do not explain the sex differences we found in the reward system.

To evaluate whether differences in the shape of the hemodynamic response might account for apparent sex differences in neural responses, we extracted the spatially averaged time-series data from the NAc for males and females in High NPY and Low NPY groups across all conditions as described by Mickey et al. (2016). Time courses were then normalized to z-scores by subtracting the mean and dividing by the standard deviation. The qualitative shape of the hemodynamic response was similar between men and women (Figure S4).

Figure S4, z-score of the hemodynamic response function of the NAc during the MID task, where women are shown in orange and men are shown in green. Subjects with the High-NPY genotype are shown on the left, and subjects with the Low-NPY genotype are shown on the right. Error bars represent standard error.

*Effects of menstrual phase*

In exploratory analyses, we tested for differences in behavioral (n=93), autonomic (n=84), and neural responses (n=19) between women estimated to be in a follicular phase (last reported period less than or equal to ten days previous) versus women estimated to be in a non-follicular phase (last reported period greater than ten days previous). We did not find any significant difference between the two groups of women (p > 0.05, Mann-Whitney test) for the salience or valence contrasts of arousal ratings, affect ratings, accuracy, SCR, or neural responses in NAc, midbrain, AI, or dACC.

*Mediation analyses*

For the sub-sample of 44 subjects with both fMRI and behavioral data available, the direct effect of sex on those outcomes was significant for arousal ratings and accuracy (p<0.05). The indirect effect of each BOLD salience contrast response was significant for accuracy (p<0.05, excepting the dorsal AI where it was trending(0.05<p<0.01), and significant for arousal ratings in the VTA, ventral AI, and NAc (p<0.05), and trending in the dACC and dorsal AI (0.05<p<0.01). For the sub-sample of 40 subjects with both fMRI and SCR data available, the direct effect of sex on SCR did not reach statistical significance (β=0.20, p=0.13), nor did the indirect effect (p>0.05). These findings indicated that BOLD response in each ROI mediated the effects of sex on behavior (Figure S5). It should be noted that the "average causal mediation effect" is a modeled parameter and cannot establish actual causality.

Figure S5, Unstandardized regression coefficients for the relationship between sex and the salience contrast of accuracy (A, C, E, G, I) and the salience contrast of arousal rating (B, D, F, H, J). The five neural mediators tested are dACC (A-B), midbrain/VTA (C-D), ventral AI (E-F), dorsal AI (G-H), and NAc (I-J) BOLD salience contrasts. See the main text for description of the mediation model. ^†^:0.05<p<0.1, *:p<0.05, **:p<0.01. ** indicates statistical significance after correction for multiple comparison.

**Table S4. Whole Brain Analyses**

**Table S5. Linear mixed model analyses of behavioral and physiological outcomes**

**

**References**

Behzadi, Y., Restom, K., Liau, J., et al. (2007). A component based noise correction method (CompCor) for BOLD and perfusion based fMRI. *NeuroImage*, **37**, 90–101

Chang, L.J., Yarkoni, T., Khaw, M.W., et al. (2013). Decoding the Role of the Insula in Human Cognition: Functional Parcellation and Large-Scale Reverse Inference. *Cerebral Cortex*, **23**, 739–49

Desikan, R.S., Ségonne, F., Fischl, B., et al. (2006). An automated labeling system for subdividing the human cerebral cortex on MRI scans into gyral based regions of interest. *NeuroImage*, **31**, 968–80

Mickey, B.J., Heffernan, J., Heisel, C., et al. (2016). Oxytocin modulates hemodynamic responses to monetary incentives in humans. *Psychopharmacology*, **233**, 3905–19

Warthen, K.G., Sanford, B., Walker, K., et al. (2018). Neuropeptide Y and representation of salience in human nucleus accumbens. *Neuropsychopharmacology*, **44**, 495–502
