# Supplementary material for: Sex differences in the human reward system: convergent behavioral, autonomic and neural evidence
Source: Soc Cogn Affect Neurosci. 2020 Jul 30;15(7):789–801. doi: 10.1093/scan/nsaa104 (PMC7511890; doi:10.1093/scan/nsaa104)
Supplement: scan-20-007-File008_nsaa104 [file scan-20-007-file008_nsaa104.docx]

| Table S1. Demographic, physiological, and clinical characteristics of the sample | | | | |  |
| --- | --- | --- | --- | --- | --- |
|  | **Imaged Subjects (n=44)** | |  | **SCR only Subjects (n=201)** | |
|  | mean | SD |  | mean | SD |
|  |  |  |  |  |  |
| Age | 20.43 | 1.24 |  | 20.43 | 1.30 |
| Female, n (%) | 24 (54.55%) |  |  | 109 (54.23%) | |
| **Race** |  |  |  |  |  |
| White, n (%) | 25 (56.82%) |  |  | 132.00 |  |
| Asian, n (%) | 7 (15.91%) |  |  | 12.00 |  |
| Black, n (%) | 2 (4.55%) |  |  | 53.00 |  |
| **Ancestry components^a^** |  |  |  |  |  |
| First component weight, w1 | 0.74 | 0.39 |  | 0.66 | 0.44 |
| Second component weight, w2 | 0.13 | 0.26 |  | 0.13 | 0.30 |
| Third component weight, w3 | 0.13 | 0.31 |  | 0.21 | 0.38 |
| **Predominant ancestry^a^** |  |  |  |  |  |
| w1 > 0.9, n (%) | 28 |  |  | 114 (57%) |  |
| w2 > 0.9, n (%) | 2 |  |  | 16 (8%) |  |
| w3 > 0.9, n (%) | 4 |  |  | 29 (14.5%) |  |
| other | 10 |  |  | 41 (20.5%) |  |
| **Physiological measures** |  |  |  |  |  |
| Heart rate (per minute)^a^ | 70.98 | 11.63 |  | 67.69 | 10.83 |
| Systolic BP (mmHg) | 113.75 | 15.8 |  | 113.33 | 13.78 |
| Diastolic BP (mmHg) | 65.66 | 9.47 |  | 63.36 | 8.81 |
| Respiratory rate (per minute)^a^ | 16.61 | 1.65 |  | 16.73 | 1.42 |
| Height (cm) | 168.93 | 8.2 |  | 170.34 | 8.91 |
| Weight (kg) | 68.96 | 16.12 |  | 69.96 | 15.62 |
| Body mass index (kg/m2) | 24.07 | 4.82 |  | 24.05 | 4.87 |
| **State measures** |  |  |  |  |  |
| PANAS positive^c^ | 30.4 | 6.06 |  | 29.20 | 7.35 |
| PANAS negative^c^ | 12.74 | 3.86 |  | 12.41 | 3.08 |
| PHQ-9^c^ | 2.86 | 2.34 |  | 3.33 | 3.61 |
| CESD^c^ | 8.29 | 5.37 |  | 8.89 | 7.74 |
| Perceived Stress Scale^c^ | 11.63 | 5.61 |  | 11.63 | 6.10 |
| Beck Anxiety Inventory^bd^ | 6.56 | 6.36 |  | 6.16 | 6.26 |
| **Trait measures** |  |  |  |  |  |
| NEO-PI-R neuroticism^a^ | 87.66 | 26.33 |  | 82.22 | 23.17 |
| NEO-PI-R extraversion^a^ | 116.32 | 22.94 |  | 123.61 | 20.03 |
| NEO-PI-R openness^a^ | 117.64 | 19.14 |  | 121.69 | 18.36 |
| NEO-PI-R agreeableness^a^ | 118.14 | 20.9 |  | 121.87 | 20.58 |
| NEO-PI-R conscientiousness^a^ | 120.84 | 21.41 |  | 122.73 | 20.22 |
| BIS-BAS behavioral inhibition^bd^ | 20.08 | 4.08 |  | 19.87 | 3.63 |
| BIS-BAS reward responsiveness^bd^ | 17.65 | 1.94 |  | 18.08 | 1.85 |
| BIS-BAS drive^bd^ | 10.7 | 2.58 |  | 11.47 | 2.67 |
| BIS-BAS fun seeking^bd^ | 2.46 | 1.94 |  | 12.09 | 2.38 |
| SPSRQ reward^bd^ | 11.93 | 3.50 |  | 11.89 | 4.00 |
| SPSRQ punishment^bd^ | 11.53 | 5.20 |  | 10.34 | 4.96 |
| Appetitive Motivation Scale^bd^ | 14.13 | 2.59 |  | 15.19 | 2.69 |
|  |  |  |  |  |  |
| PANAS: Positive and Negative Affect Schedule | |  |  |  |  |
| CESD: Center for Epidemiologic Studies Depression Scale | | |  |  |  |
| NEO-PI-R: Neuroticism, Extraversion, Openness Personality Inventory - Revised | | | | | |
| BIS-BAS: Behavioral Inhibition and Approach Scales | |  |  |  |  |
| SPSRQ: Sensitivity to Punishment and Sensitivity to Reward Questionnaire | | | | |  |
| a: Missing data for 1-2 SCR only subjects | |  |  |  |  |
| b: Missing data for 6-8 SCR only subjects | |  |  |  |  |
| c: Missing data for 1 imaged subject |  |  |  |  |  |
| d: Missing data for 3-4 imaged subjects | |  |  |  |  |
