# Supplementary material for: Sex differences in the human reward system: convergent behavioral, autonomic and neural evidence
Source: Soc Cogn Affect Neurosci. 2020 Jul 30;15(7):789–801. doi: 10.1093/scan/nsaa104 (PMC7511890; doi:10.1093/scan/nsaa104)
Supplement: scan-20-007-File009_nsaa104 [file scan-20-007-file009_nsaa104.docx]

| Table S2. Additional demographic and clinical characteristics of the sample | |  |  |  |  |  |  |  |  |
| --- | --- | --- | --- | --- | --- | --- | --- | --- | --- |
|  | **All (n=221)** |  |  | **Men (n=100)** |  |  | **Women (n=121)** | |  |
|  | mean | SD or % |  | mean | SD or % |  | mean | SD |  |
|  |  |  |  |  |  |  |  |  |  |
| **Ancestry components^a^** |  |  |  |  |  |  |  |  |  |
| First component weight, w1 | 0.64 | 0.44 |  | 0.66 | 0.44 |  | 0.61 | 0.44 |  |
| Second component weight, w2 | 0.14 | 0.31 |  | 0.16 | 0.33 |  | 0.13 | 0.28 |  |
| Third component weight, w3 | 0.22 | 0.39 |  | 0.18 | 0.37 |  | 0.26 | 0.40 |  |
| **MINI^b^** |  |  |  |  |  |  |  |  |  |
| Current Depression | 4 | 1.8% |  | 2 | 2.0% |  | 2 | 1.7% |  |
| Past Depression | 31 | 14.0% |  | 10 | 10.0% |  | 21 | 17.4% |  |
| Current Dysthymia | 1 | 0.5% |  | 0 | 0.0% |  | 1 | 0.8% |  |
| Past Dysthymia | 1 | 0.5% |  | 1 | 1.0% |  | 0 | 0.0% |  |
| Past Hypomania or Mania | 5 | 2.3% |  | 1 | 1.0% |  | 4 | 3.3% |  |
| Current Agoraphobia | 8 | 3.6% |  | 3 | 3.0% |  | 5 | 4.1% |  |
| Current Social Phobia | 11 | 5.0% |  | 1 | 1.0% |  | 10 | 8.3% | * |
| Current Obsessive Compulsive Disorder | 2 | 0.9% |  | 1 | 1.0% |  | 1 | 0.8% |  |
| Alcohol Dependence^c^ | 9 | 4.1% |  | 4 | 4.0% |  | 5 | 4.1% |  |
| Alcohol Abuse^c^ | 7 | 3.2% |  | 5 | 5.0% |  | 2 | 1.7% |  |
| Psychoactive Substance Use Dependence^c^ | 2 | 0.9% |  | 2 | 2.0% |  | 0 | 0.0% |  |
| Psychoactive Substance Use Abuse^c^ | 3 | 1.4% |  | 2 | 2.0% |  | 1 | 0.8% |  |
| Current Tobacco use | 10 | 4.5% |  | 6 | 6.0% |  | 4 | 3.3% |  |
| Current Mood Disorder with Psychotic Features | 1 | 0.5% |  | 1 | 1.0% |  | 0 | 0.0% |  |
| Current Bulimia Nervosa | 2 | 0.9% |  | 0 | 0.0% |  | 2 | 1.7% |  |
| Current Generalized Anxiety Disorder | 10 | 4.5% |  | 0 | 0.0% |  | 10 | 8.3% | * |
| Adult Attention Deficit/Hyperactivity Disorder | 5 | 2.3% |  | 4 | 4.0% |  | 1 | 0.8% |  |
| MINI: MINI International Neuropsychiatric Interview |  |  |  |  |  |  |  |  |  |
| a: n=220 |  |  |  |  |  |  |  |  |  |
| b: No incidence reported in the MINI for: mood disorder due to a medical condition (past or present), current hypomanic episode, current manic episode, current panic disorder, post-truamatic stress disorder, psychotic disorder (current or lifetime), anorexia, past mood disorder with psychotic features, or lifetime psychotic disorder | | | | | | | | |  |
|  |  |  |  |  |  |  |  |  |  |
| c: Criteria met within the past twelve months |  |  |  |  |  |  |  |  |  |
| *: p<0.05, Fisher exact test, male vs. female |  |  |  |  |  |  |  |  |  |
