# Supplementary material for: Sex differences in the human reward system: convergent behavioral, autonomic and neural evidence
Source: Soc Cogn Affect Neurosci. 2020 Jul 30;15(7):789–801. doi: 10.1093/scan/nsaa104 (PMC7511890; doi:10.1093/scan/nsaa104)
Supplement: scan-20-007-File010_nsaa104 [file scan-20-007-file010_nsaa104.docx]

| Table S3. Demographic, physiological, and clinical characteristics of the imaged sample, male versus female | | | | | | |
| --- | --- | --- | --- | --- | --- | --- |
|  | **Male Imaged Subjects (n=20)** | |  | **Female Imaged Subjects (n=24)** | |  |
|  | mean | SD |  | mean | SD |  |
| Age | 20.63 | 1.35 |  | 20.27 | 1.13 |  |
| **Race** |  |  |  |  |  |  |
| White, n (%) | 17 (85%) |  |  | 18.00 |  |  |
| Asian, n (%) | 3 (15%) |  |  | 4.00 |  |  |
| Black, n (%) | 0.00 |  |  | 2.00 |  |  |
| **Ancestry components** |  |  |  |  |  |  |
| First component weight, w1 | 0.80 | 0.36 |  | 0.70 | 0.41 |  |
| Second component weight, w2 | 0.20 | 0.35 |  | 0.07 | 0.15 |  |
| Third component weight, w3 | 0.008 | 0.03 |  | 0.23 | 0.39 |  |
| **Predominant ancestry** |  |  |  |  |  |  |
| w1 > 0.9, n (%) | 14 |  |  | 14 |  |  |
| w2 > 0.9, n (%) | 2 |  |  | 0 |  |  |
| w3 > 0.9, n (%) | 0 |  |  | 4 |  |  |
| other | 4 |  |  | 6 |  |  |
| **Physiological measures** |  |  |  |  |  |  |
| Heart rate (per minute) | 68.05 | 12.41 |  | 73.42 | 10.57 |  |
| Systolic BP (mmHg) | 119.00 | 16.95 |  | 109.38 | 13.62 | * |
| Diastolic BP (mmHg) | 66.55 | 12.20 |  | 64.92 | 6.61 |  |
| Respiratory rate (per minute) | 16.75 | 1.48 |  | 16.5 | 1.79 |  |
| Height (cm) | 174.45 | 7.33 |  | 164.34 | 5.74 | * |
| Weight (kg) | 74.12 | 15.96 |  | 64.66 | 15.26 | * |
| Body mass index (kg/m2) | 24.26 | 4.41 |  | 23.91 | 5.23 |  |
| **State measures** |  |  |  |  |  |  |
| PANAS positive^a^ | 29.40 | 6.11 |  | 31.26 | 6.01 |  |
| PANAS negative^a^ | 12.50 | 3.55 |  | 12.96 | 4.18 |  |
| PHQ-9^a^ | 3.50 | 3.20 |  | 2.87 | 2.42 |  |
| CESD^a^ | 7.70 | 6.71 |  | 7.87 | 5.98 |  |
| Perceived Stress Scale^a^ | 10.85 | 6.24 |  | 12.35 | 6.41 |  |
| Beck Anxiety Inventory^b^ | 5.89 | 5.91 |  | 5.87 | 5.98 |  |
| **Trait measures** |  |  |  |  |  |  |
| NEO-PI-R neuroticism | 82.70 | 26.79 |  | 91.79 | 25.77 |  |
| NEO-PI-R extraversion | 118.70 | 19.72 |  | 114.33 | 25.57 |  |
| NEO-PI-R openness | 113.70 | 19.50 |  | 120.92 | 18.61 |  |
| NEO-PI-R agreeableness | 114.65 | 18.64 |  | 121.04 | 22.59 |  |
| NEO-PI-R conscientiousness | 116.70 | 19.95 |  | 124.29 | 22.39 |  |
| BIS-BAS behavioral inhibition^b^ | 19.11 | 4.29 |  | 20.95 | 3.77 |  |
| BIS-BAS reward responsiveness^b^ | 17.26 | 2.31 |  | 18.00 | 1.52 |  |
| BIS-BAS drive^b^ | 10.58 | 2.71 |  | 10.81 | 2.52 |  |
| BIS-BAS fun seeking^b^ | 12.16 | 2.24 |  | 10.71 | 2.49 | * |
| SPSRQ reward^b^ | 12.47 | 3.79 |  | 11.43 | 3.23 |  |
| SPSRQ punishment^b^ | 10.84 | 5.68 |  | 12.14 | 4.78 |  |
| Appetitive Motivation Scale^b^ | 14.21 | 2.37 |  | 14.05 | 2.84 |  |
| **MINI^c^** |  |  |  |  |  |  |
| Past Depression | 1 |  |  | 4 |  |  |
| Past Hypomania or Mania | 0 |  |  | 1 |  |  |
| Current Social Phobia | 0 |  |  | 2 |  |  |
| Alcohol Abuse | 1 |  |  | 0 |  |  |
| Current Tobacco use | 1 |  |  | 1 |  |  |
| Adult Attention Deficit/Hyperactivity Disorder | 1 |  |  | 0 |  |  |
| PANAS: Positive and Negative Affect Schedule | |  |  |  |  |  |
| CESD: Center for Epidemiologic Studies Depression Scale | | |  |  |  |  |
| NEO-PI-R: Neuroticism, Extraversion, Openness Personality Inventory - Revised | | | | |  |  |
| BIS-BAS: Behavioral Inhibition and Approach Scales | | |  |  |  |  |
| SPSRQ: Sensitivity to Punishment and Sensitivity to Reward Questionnaire | | | | |  |  |
| MINI: MINI International Neuropsychiatric Interview | | |  |  |  |  |
| b: Missing data for 1 male and 2-3 female subjects | | |  |  |  |  |
| c: No incidence reported in the MINI for: current depression, current or past dysthymia, current agoraphobia, mood disorder due to a medical condition (past or present), current hypomanic episode, current manic episode, current panic disorder, post-truamatic stress disorder, psychotic disorder (current or lifetime), current obsessive compulsive disorder, alcohold dependence, psychoactive substance use dependence or abuse, anorexia, bulimia, current generalized anxiety disorder, current or past mood disorder with psychotic features, or lifetime psychotic disorder | | | | | | |
|  |  |  |  |  |  |  |
| *: p<0.05 Mann-Whitney test, male vs. female | |  |  |  |  |  |
