# Supplementary material for: Sex differences in the human reward system: convergent behavioral, autonomic and neural evidence
Source: Soc Cogn Affect Neurosci. 2020 Jul 30;15(7):789–801. doi: 10.1093/scan/nsaa104 (PMC7511890; doi:10.1093/scan/nsaa104)
Supplement: scan-20-007-File011_nsaa104 [file scan-20-007-file011_nsaa104.docx]

| Table S4. Whole brain analyses | | |  |  |  |  |  |  |  |
| --- | --- | --- | --- | --- | --- | --- | --- | --- | --- |
| High Salience vs. Low Salience | | |  |  |  |  |  |  |  |
| **Men>Women** | |  |  |  |  |  |  |  |  |
| Statistics: p-values adjusted for search volume | | | |  |  |  |  |  |  |
| cluster-level | cluster-level | cluster-level | cluster-level | peak-level | peak-level | peak-level | peak-level | peak-level |  |
| p(FWE-corr) | p(FDR-corr) | equivk | p(unc) | p(FWE-corr) | p(FDR-corr) | T | equivZ | p(unc) | x,y,z {mm} |
| 0.664 | 0.66 | 17 | 0.149 | 0.537 | 0.759 | 4.40 | 3.90 | 0.000 | (-39, -13, -26) |
| 0.534 | 0.66 | 22 | 0.104 | 0.909 | 0.759 | 3.89 | 3.52 | 0.000 | (-60, -31, 34) |
|  |  |  |  | 0.973 | 0.759 | 3.70 | 3.38 | 0.000 | (-66, -37, 28) |
| table shows 3 local maxima more than 8.0mm apart | | | |  |  |  |  |  |  |
| Height threshold: T = 3.34, p = 0.001 (0.999) | | | |  |  |  |  |  |  |
| Extent threshold: k = 10 voxels, p = 0.263 (0.854) | | | |  |  |  |  |  |  |
|  |  |  |  |  |  |  |  |  |  |
| **Women>Men** | |  |  |  |  |  |  |  |  |
| no supra threshold clusters | | |  |  |  |  |  |  |  |
| Height threshold: T = 3.34, p = 0.001 (0.999) | | | |  |  |  |  |  |  |
| Extent threshold: k = 10 voxels, p = 0.263 (0.854) | | | |  |  |  |  |  |  |
|  |  |  |  |  |  |  |  |  |  |
| Positive Valence vs. Negative Valence | | |  |  |  |  |  |  |  |
| **Men>Women** | |  |  |  |  |  |  |  |  |
| Statistics: p-values adjusted for search volume | | | |  |  |  |  |  |  |
| cluster-level | cluster-level | cluster-level | cluster-level | peak-level | peak-level | peak-level | peak-level | peak-level |  |
| p(FWE-corr) | p(FDR-corr) | equivk | p(unc) | p(FWE-corr) | p(FDR-corr) | T | equivZ | p(unc) | x,y,z {mm} |
| 0.568 | 0.669 | 18 | 0.084 | 0.607 | 0.468 | 4.46 | 3.94 | 0 | (-18, -40, 52) |
|  |  |  |  | 0.989 | 0.812 | 3.74 | 3.41 | 0 | (-21, -31, 49) |
| table shows 3 local maxima more than 8.0mm apart | | | |  |  |  |  |  |  |
| Height threshold: T = 3.34, p = 0.001 (1.000) | | | |  |  |  |  |  |  |
| Extent threshold: k = 10 voxels, p = 0.187 (0.847) | | | |  |  |  |  |  |  |
|  |  |  |  |  |  |  |  |  |  |
| **Women>Men** | |  |  |  |  |  |  |  |  |
| Statistics: p-values adjusted for search volume | | | |  |  |  |  |  |  |
| cluster-level | cluster-level | cluster-level | cluster-level | peak-level | peak-level | peak-level | peak-level | peak-level |  |
| p(FWE-corr) | p(FDR-corr) | equivk | p(unc) | p(FWE-corr) | p(FDR-corr) | T | equivZ | p(unc) | x,y,z {mm} |
| 0.847 | 0.21 | 10 | 0.187 | 0.866 | 0.241 | 4.12 | 3.69 | 0 | (27, -85, 31) |
| table shows 3 local maxima more than 8.0mm apart | | | |  |  |  |  |  |  |
| Height threshold: T = 3.34, p = 0.001 (1.000) | | | |  |  |  |  |  |  |
| Extent threshold: k = 10 voxels, p = 0.187 (0.847) | | | |  |  |  |  |  |  |
|  |  |  |  |  |  |  |  |  |  |
