# Supplementary material for: Sex differences in the human reward system: convergent behavioral, autonomic and neural evidence
Source: Soc Cogn Affect Neurosci. 2020 Jul 30;15(7):789–801. doi: 10.1093/scan/nsaa104 (PMC7511890; doi:10.1093/scan/nsaa104)
Supplement: scan-20-007-File012_nsaa104 [file scan-20-007-file012_nsaa104.docx]

| Table S5. Linear mixed model analyses of behavioral and physiological outcomes | | | | |
| --- | --- | --- | --- | --- |
| Task Accuracy (n=221) | | |  |  |
|  | chi-squared | p-value |  |  |
| Main effect of sex | 0.037 | 0.85 |  |  |
| Main effect of valence | 8.24 | 4.10E-03 |  |  |
| Main effect of salience | 626.62 | 2.20E-16 |  |  |
| Sex*Valence | 0.18 | 0.67 |  |  |
| Sex*Salience | 14.84 | 0.00012 |  |  |
|  |  |  |  |  |
| Arousal Rating (n=221) | | |  |  |
|  | chi-squared | p-value |  |  |
| Main effect of sex | 1.35 | 0.24 |  |  |
| Main effect of valence | 209.3 | 2.20E-16 |  |  |
| Main effect of salience | 1085.84 | 2.20E-16 |  |  |
| Sex*Valence | 0.88 | 0.35 |  |  |
| Sex*Salience | 14.14 | 0.00017 |  |  |
|  |  |  |  |  |
| Affect Rating (n=221) | | |  |  |
|  | chi-squared | p-value |  |  |
| Main effect of sex | 0.0004 | 0.98 |  |  |
| Main effect of valence | 2242.61 | <2e-16 |  |  |
| Main effect of salience | 92.99 | <2e-16 |  |  |
| Sex*Valence | 0.38 | 0.54 |  |  |
| Sex*Salience | 0.1198 | 0.73 |  |  |
|  |  |  |  |  |
| SCR (n=201) | | |  |  |
|  | chi-squared | p-value |  |  |
| Main effect of sex | 2.94 | 0.086 |  |  |
| Main effect of valence | 1.89 | 0.17 |  |  |
| Main effect of salience | 439.97 | 2.20E-16 |  |  |
| Sex*Valence | 0.12 | 0.73 |  |  |
| Sex*Salience | 19.89 | 8.21E-06 |  |  |
|  |  |  |  |  |
| NAc Salience Contrast (n=44) | | |  |  |
|  | chi-squared | p-value |  |  |
| Main effect of sex | 4.88 | 0.027 |  |  |
| Main effect of valence | 102.59 | 2.20E-16 |  |  |
| Main effect of salience | 761.25 | 2.20E-16 |  |  |
| Sex*Valence | 4.096 | 0.043 |  |  |
| Sex*Salience | 85.17 | 2.20E-16 |  |  |
|  |  |  |  |  |
| dAcc Salience Contrast (n=44) | | |  |  |
|  | chi-squared | p-value |  |  |
| Main effect of sex | 9.15 | 2.50E-03 |  |  |
| Main effect of valence | 24.49 | 7.47E-07 |  |  |
| Main effect of salience | 537.90 | 2.20E-16 |  |  |
| Sex*Valence | 11.086 | 0.00087 |  |  |
| Sex*Salience | 87.015 | 2.20E-16 |  |  |
|  |  |  |  |  |
| Ventral AI Salience Contrast (n=44) | | |  |  |
|  | chi-squared | p-value |  |  |
| Main effect of sex | 4.035 | 4.50E-02 |  |  |
| Main effect of valence | 8.01 | 0.0047 |  |  |
| Main effect of salience | 183.46 | 2.20E-16 |  |  |
| Sex*Valence | 2.51 | 0.11 |  |  |
| Sex*Salience | 49.71 | 1.79E-12 |  |  |
|  |  |  |  |  |
| Dorsal AI Salience Contrast (n=44) | | |  |  |
|  | chi-squared | p-value |  |  |
| Main effect of sex | 4.27 | 0.038 |  |  |
| Main effect of valence | 2.56 | 0.11 |  |  |
| Main effect of salience | 240.96 | 2.20E-16 |  |  |
| Sex*Valence | 0.76 | 0.38 |  |  |
| Sex*Salience | 56.56 | 5.46E-14 |  |  |
|  |  |  |  |  |
| Midbrain Salience Contrast (n=44) | | |  |  |
|  | chi-squared | p-value |  |  |
| Main effect of sex | 0.32 | 0.57 |  |  |
| Main effect of valence | 15.21 | 9.62E-05 |  |  |
| Main effect of salience | 585.86 | 2.20E-16 |  |  |
| Sex*Valence | 1.15 | 0.28 |  |  |
| Sex*Salience | 61.43 | 4.60E-15 |  |  |
