# Supplementary figures and images for: Sex differences in the human reward system: convergent behavioral, autonomic and neural evidence
Source: Soc Cogn Affect Neurosci. 2020 Jul 30;15(7):789–801. doi: 10.1093/scan/nsaa104 (PMC7511890; doi:10.1093/scan/nsaa104)

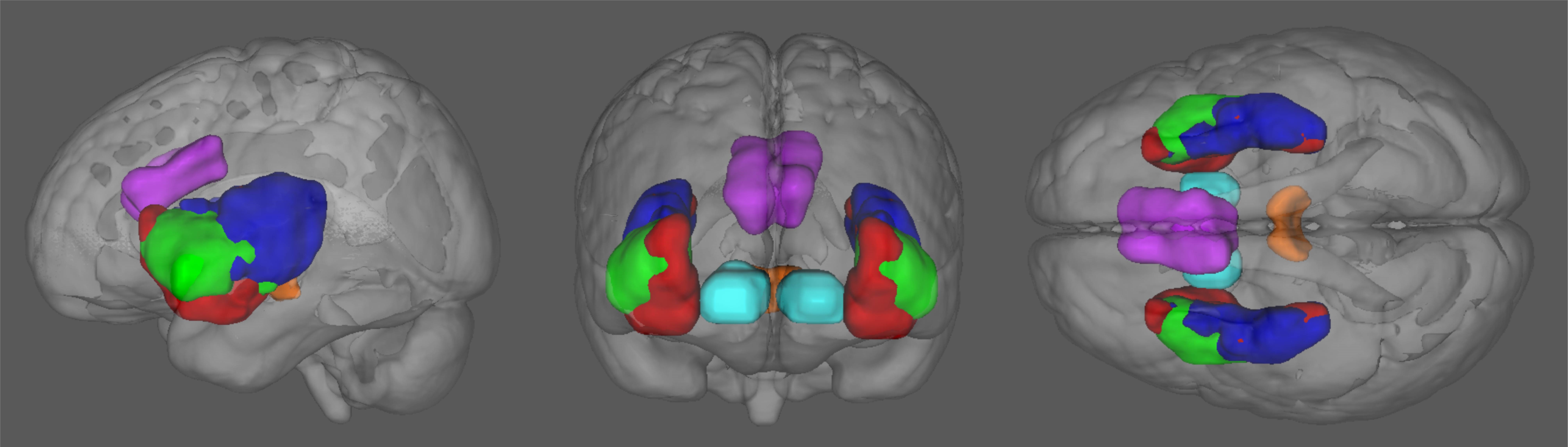

Supplement: scan-20-007-File013_nsaa104 [file scan-20-007-file013_nsaa104.jpeg]

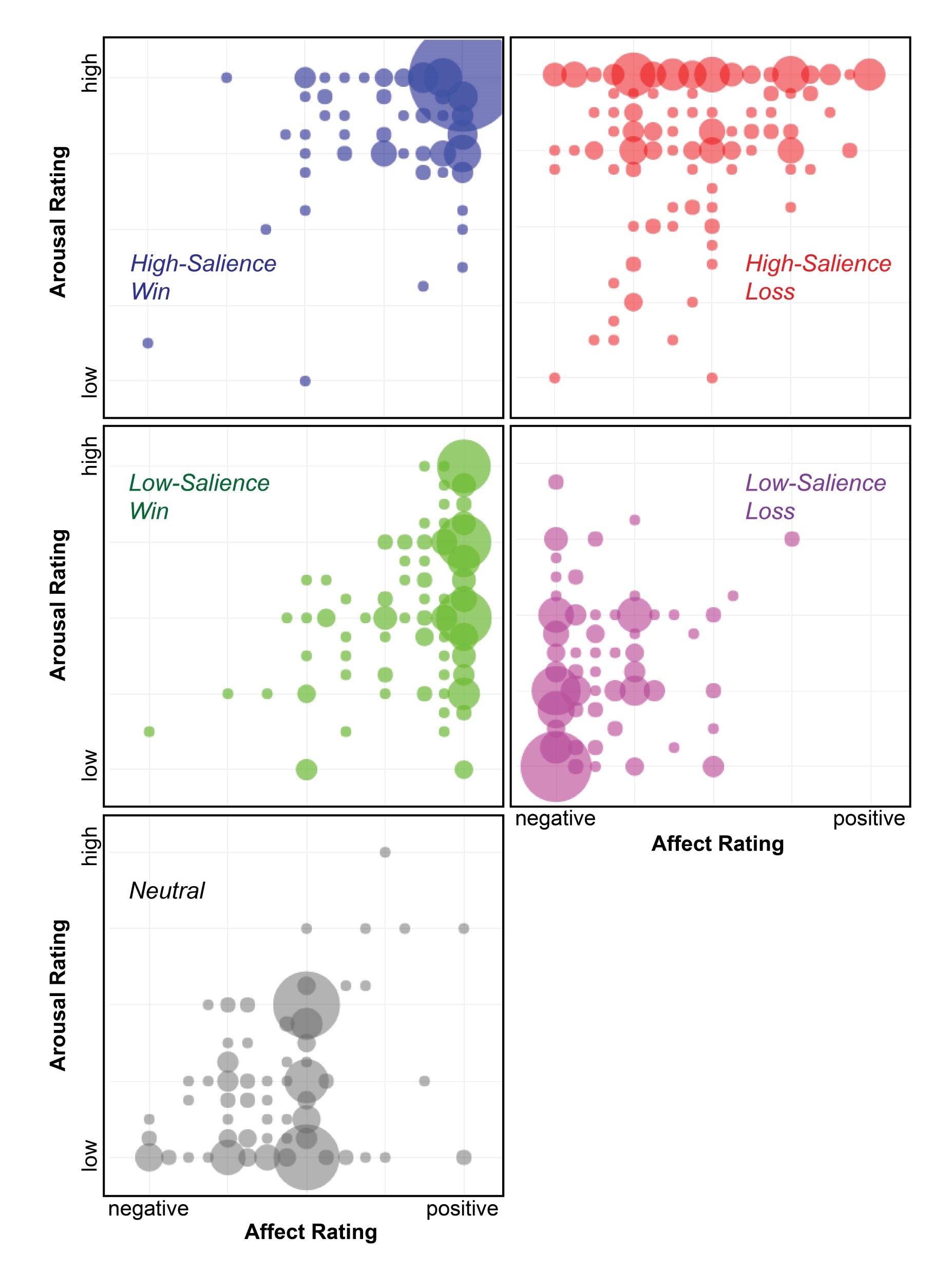

Supplement: scan-20-007-File014_nsaa104 [file scan-20-007-file014_nsaa104.jpeg]

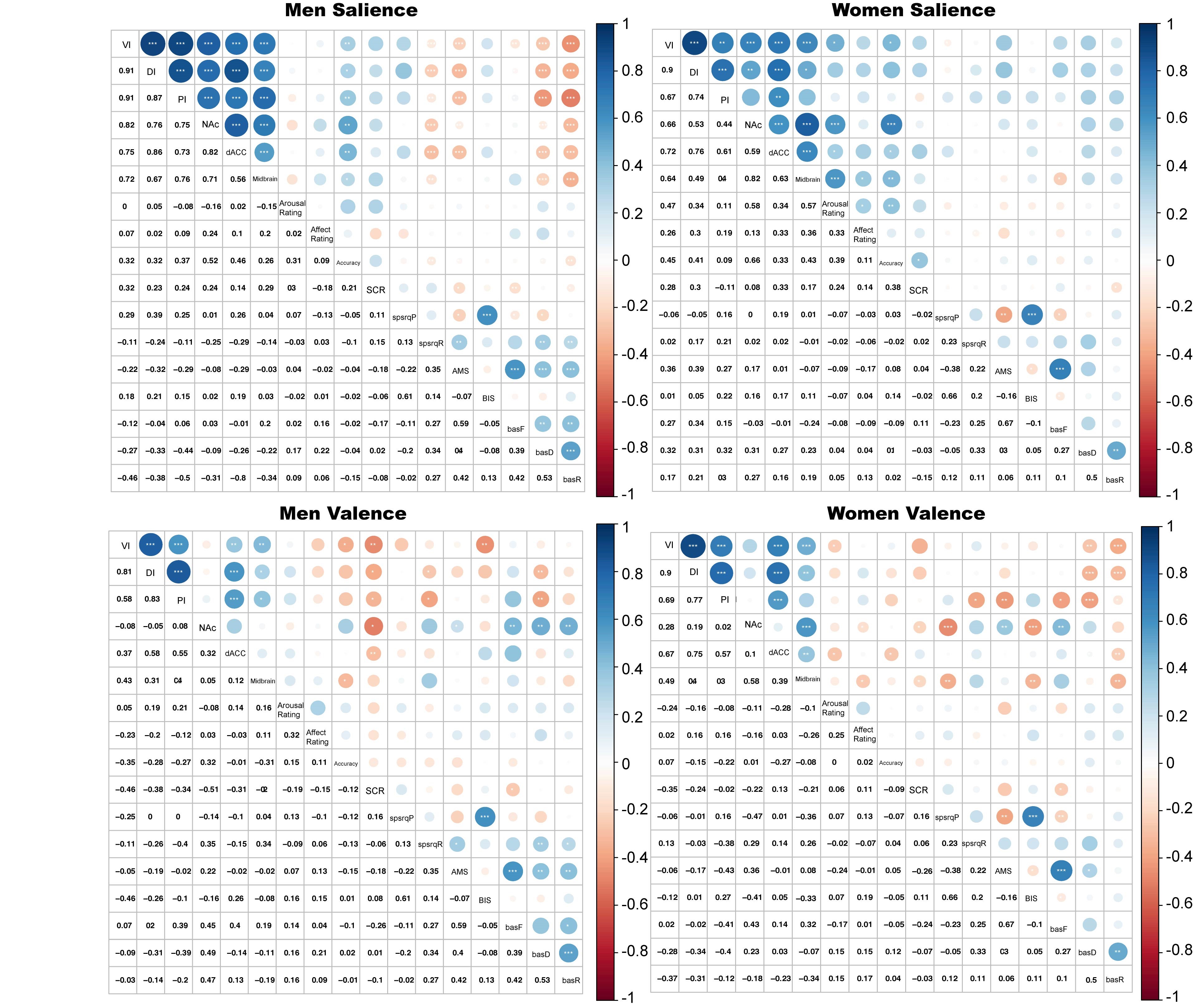

Supplement: scan-20-007-File015_nsaa104 [file scan-20-007-file015_nsaa104.jpeg]

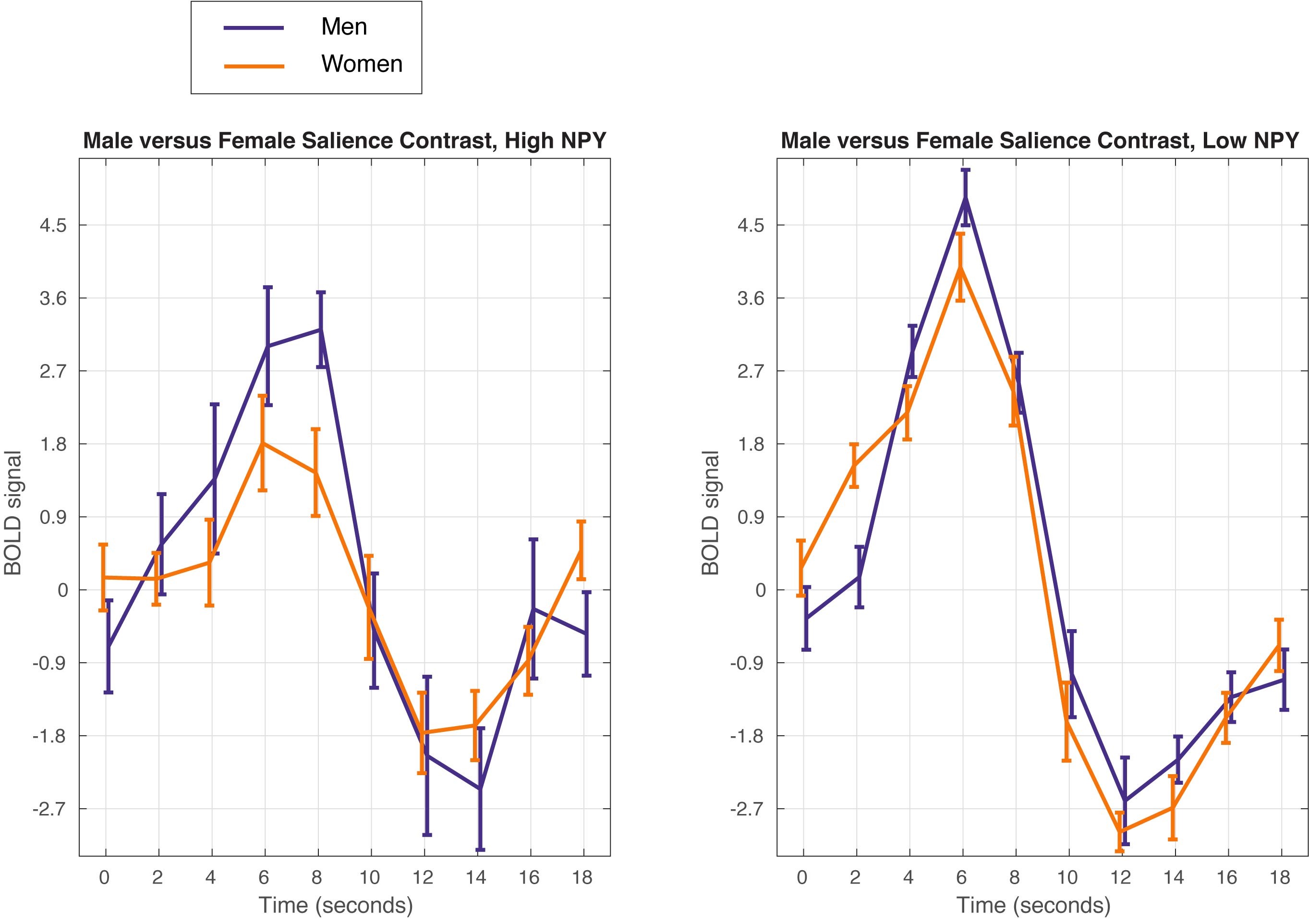

Supplement: scan-20-007-File016_nsaa104 [file scan-20-007-file016_nsaa104.jpeg]

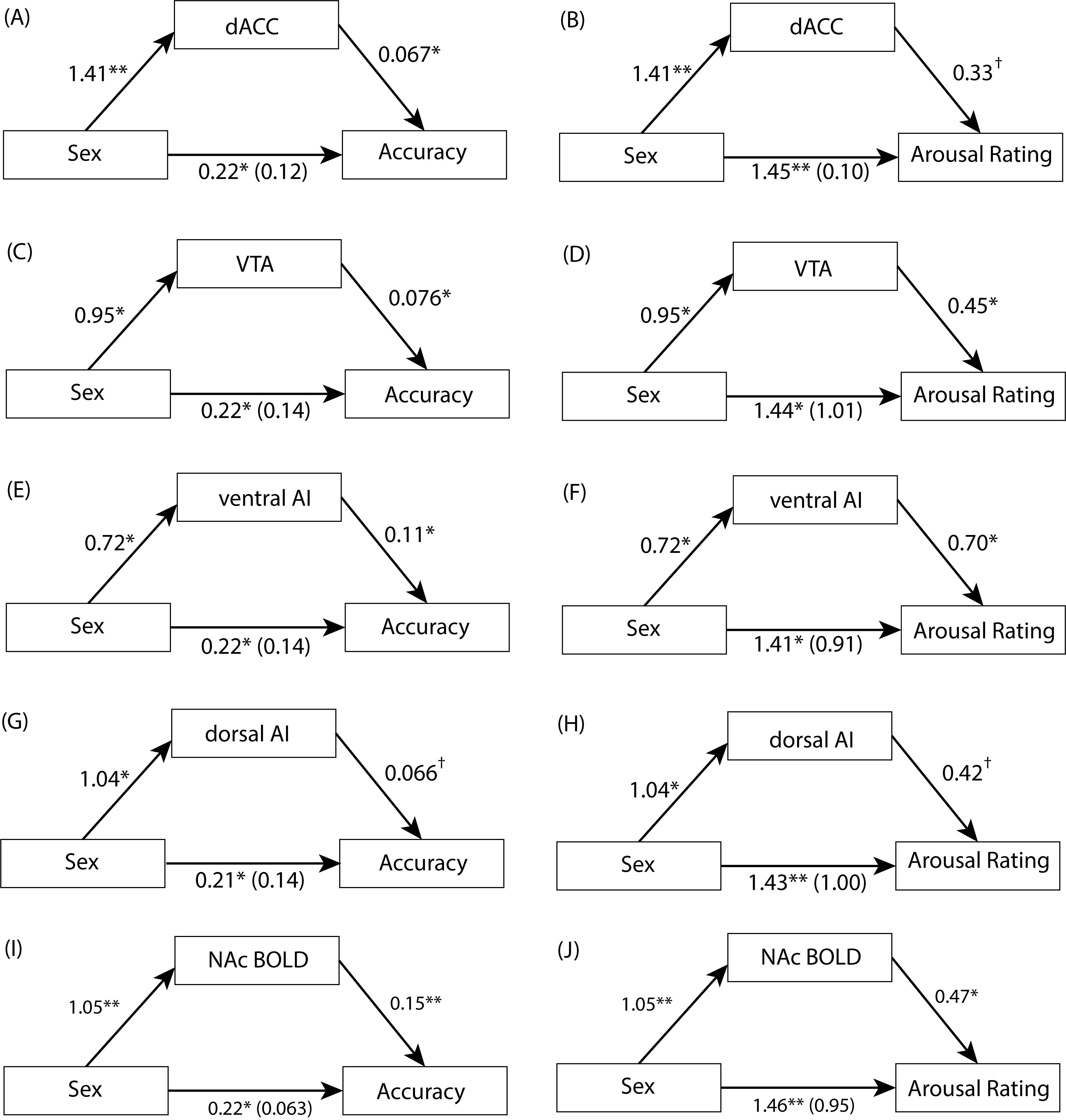

Supplement: scan-20-007-File017_nsaa104 [file scan-20-007-file017_nsaa104.jpeg]
